# Averting HIV Infections in New York City: A Modeling Approach Estimating the Future Impact of Additional Behavioral and Biomedical HIV Prevention Strategies

**DOI:** 10.1371/journal.pone.0073269

**Published:** 2013-09-13

**Authors:** Jason Kessler, Julie E. Myers, Kimberly A. Nucifora, Nana Mensah, Alexis Kowalski, Monica Sweeney, Christopher Toohey, Amin Khademi, Colin Shepard, Blayne Cutler, R. Scott Braithwaite

**Affiliations:** 1 Division of Comparative Effectiveness and Decision Science, Department of Population Health, New York University School of Medicine, New York, New York, United States of America; 2 Bureau of HIV/AIDS Prevention and Control, New York City Department of Health and Mental Hygiene, New York, New York, United States of America; 3 Division of Infectious Diseases, Columbia University Medical Center, New York, New York, United States of America; 4 Department of Industrial Engineering, University of Pittsburgh, Pittsburgh, Pennsylvania, United States of America; Yale School of Public Health, United States of America

## Abstract

**Background:**

New York City (NYC) remains an epicenter of the HIV epidemic in the United States. Given the variety of evidence-based HIV prevention strategies available and the significant resources required to implement each of them, comparative studies are needed to identify how to maximize the number of HIV cases prevented most economically.

**Methods:**

A new model of HIV disease transmission was developed integrating information from a previously validated micro-simulation HIV disease progression model. Specification and parameterization of the model and its inputs, including the intervention portfolio, intervention effects and costs were conducted through a collaborative process between the academic modeling team and the NYC Department of Health and Mental Hygiene. The model projects the impact of different prevention strategies, or portfolios of prevention strategies, on the HIV epidemic in NYC.

**Results:**

Ten unique interventions were able to provide a prevention benefit at an annual program cost of less than $360,000, the threshold for consideration as a cost-saving intervention (because of offsets by future HIV treatment costs averted). An optimized portfolio of these specific interventions could result in up to a 34% reduction in new HIV infections over the next 20 years. The cost-per-infection averted of the portfolio was estimated to be $106,378; the total cost was in excess of $2 billion (over the 20 year period, or approximately $100 million per year, on average). The cost-savings of prevented infections was estimated at more than $5 billion (or approximately $250 million per year, on average).

**Conclusions:**

Optimal implementation of a portfolio of evidence-based interventions can have a substantial, favorable impact on the ongoing HIV epidemic in NYC and provide future cost-saving despite significant initial costs.

## Introduction

New York City (NYC) remains an epicenter of HIV in the U.S. More than 110,000 New Yorkers are living with HIV, and almost 3,500 new cases of HIV were diagnosed in 2010 [1]. NYC's AIDS case rate is almost 3 times the U.S. average, and HIV is the third leading cause of death for NYC residents aged 35 to 54 [2]. While no single prevention strategy has materialized to control the HIV epidemic, a number of behavioral and biomedical approaches have been developed that reduce the risk of HIV infection [3]. In fact, some investigators have theorized that the HIV epidemic can even be extinguished in certain settings with systematic prioritization and implementation of a package of aggressive interventions (e.g., universal annual testing, prompt linkage to care, and immediate antiretroviral therapy [ART] initiation) [4]. However, these interventions require substantial resources, and it remains unclear how to best allocate HIV prevention resources to maximize the number of new HIV cases prevented. Furthermore, detection and care patterns in the US differ considerably from the optimistic assumptions of recent models [4].

While the National HIV/AIDS Strategy (preventing new infections, increasing access to care, and reducing HIV-related health disparities) [5] and the new focus of CDC's ”High-Impact Prevention” (HIP) (intensifying the use of appropriately combined evidence-based prevention methods in the most highly affected geographic areas) [6] may provide new momentum to HIV prevention efforts in the United States, additional tools to prioritize and focus currently available prevention intervention strategies are clearly needed.

Previous modeling work comparing variegated HIV prevention interventions and strategies has been relatively scarce to date and has been associated with several limitations. For example, while a cost-effectiveness analysis of HIV prevention interventions was particularly helpful because it enabled interventions to be rank-ordered by absolute benefit and cost-per-infection averted, [7] it did not permit decision makers to individualize results based on the strength and quality of the evidence (e.g., controlled trial based data vs. observational data). Additionally, a similar model has evaluated prevention strategies from a nationwide perspective which may not account for jurisdiction-level differences in transmission dynamics, cost, and political feasibility of various interventions, all of which can contribute to local micro-epidemics and require setting-specific solutions [8].

We created a jurisdiction-specific operations research model of HIV prevention in NYC to account for complexities and local dynamics inherent in HIV transmission and treatment. This model deployed the set of evidence-based HIV prevention interventions outlined in the 2010 Center for Disease Control's Enhanced Comprehensive HIV Prevention Planning (ECHPP) grant, Phase I (see Table 1) [9]. The aims of this project were to inform HIV prevention planning in the jurisdiction by comparing cost-per-infection averted between the various ECHPP strategies and by identifying the optimal package of prevention services in NYC.

**Table 1:** HIV prevention interventions and associated costs considered in transmission simulation.

Abbreviation	ECHPP Intervention description	Cost range considered^1^	Level of Evidence^2^
Testing – clinical	Enhanced routine opt-out screening for clinical settings	$37–147	B
Testing – non-clinical	HIV testing in non-clinical settings to identify undiagnosed HIV infection	$109–162	B
Condom distribution	Condom distribution prioritized to specific populations	$0.05–$1.00	A
Post-exposure prophylaxis (PEP)^3^	Provision of Post-Exposure Prophylaxis to populations	$1312–$3938	C
Linkage to care	Implement linkage to HIV care, treatment, and prevention services for those testing HIV positive and not currently in care	$1078–$1424	B
Care coordination	Implement interventions or strategies promoting adherence to antiretroviral medication and retention in care for HIV-positive persons	$3000–$9000	B
STD	Implement STD screening according to current guidelines for specific populations	$178–230	D
Partner services	Implement ongoing partner services for HIV-positive persons (i.e., provision of partner services both at the time of diagnosis and as needed thereafter)	$748–2244	B
Risk reduction	Behavioral risk screening followed by risk reduction interventions for HIV-positive persons (including those for HIV-discordant couples) at risk of transmitting HIV	$1000–2813	D
Linkage to support services	Implement linkage to other medical and social services for HIV-positive persons	$398–1194	D
Social marketing	HIV and sexual health communication or social marketing campaigns targeted to relevant audiences	$4–13	D
Community-level evidence based interventions	Evidence based community interventions that reduce HIV risk	$0.37–$1.10	D
Prioritized use of surveillance data	Targeted use of HIV and STD surveillance data to prioritize risk reduction counseling and partner services for persons with previously diagnosed HIV infection with a new STD	$52–157	D
Social services	For HIV-negative persons at highest risk , linkages to social support services impacting HIV incidence	$88–263	D
Screening, brief intervention, and referral to treatment for unhealthy alcohol users (SBIRT)	Brief alcohol screening, interventions and referral to treatment	$55–156	C
Cofactors	Brief screening and treatment for comorbid STDs, substance use and mental health.	$55–156	C

1 For all interventions shown (with the exception of linkage to care), cost ranges considered reflect the cost in 2010 USD for each prioritized individual based on actual or estimated programmatic costs incurred by NYC DOHMH. For linkage to care, cost estimate comes from Gardner LI, et al. 2005 [25].

2 Level of evidence assignment reflects weakest evidence for a specific intervention's effects on pathway(s).

3 Includes cost of medications required.

## Methods

### Overview

An operations research model was constructed to inform HIV prevention decisions in NYC. This model incorporates information from an individual-based, stochastic simulation of HIV progression into a deterministic epidemic model of HIV transmission. The simulation estimates the HIV epidemic over varying time horizons up to 20 years. Different combinations of prevention strategies (“packages”) were tested. Costs were estimated on an incremental basis in 2010 US dollars. Benefits were measured as number and percentage of infections averted (as compared to the base case). Cost-per-infection averted ratios were determined for each package, uncertainty bounds around each estimate were created by evaluating each intervention using the lower and upper bounds of efficacy considered (Table 2). Key model parameters were varied in sensitivity analysis. For the purposes of this analysis, a threshold of $360,000 per infection averted was selected as cost-saving, since the downstream medical costs averted from preventing a new infection would offset the programmatic costs of preventing that new infection [10].

**Table 2:** Intervention-pathway effect parameter inputs into ECHPP HIV epidemic computer simulation.

Intervention→pathway effects	Effect Size^1^	Sensitivity Analysis limits	Reference
Condom distribution/use	12.3% increase (RR ∼1.12)	3.3–21.5%	Charania et al 2010 [26]
Enhanced clinic based HIV testing	32.7% increase (RR∼1.33)	29.6–39.5%	Anaya et al 2008 [27], Calderon et al 2011 [28], Mullins et al 2010 [29]
Community based HIV testing	10.2% increase (RR∼1.10)	8.0–18.9%	Rhodes et al 2011 [30], Wilton et al 2009 [31]
PEP utilization	42.0% increase (RR ∼1.42)	25.0–70.0%	Barash et al 2010 [32]
Linkage to care	30.0% increase (RR∼1.30)	9.0–37.5%	Gardner et al 2005 [25], [33]
Care coordination/Case management	20.0% increase (RR∼1.20)	7.5–32.0%	Hart et al 2010 [34], Simoni et al 2006 [35]
STD care and treatment	28.0% decrease (RR∼0.72)	8.0–51.0%	Grosskurth H et al 1995 [36]
SBIRT component effect size^2^	15.0% decrease (RR∼0.85)	5.0–25.0%	Bertholet N et al 2005 [37]
Partner services intervention^3^	2.8% increase	2.0–5.0%	Hogben et al 2007 [38], unpublished data from NYC DOH
IDU risk reduction	67.4% decrease (RR ∼0.33)	15.2–88.5%	Latkin et al 2003 [39], Robles et al 2004 [40]
Risky sexual practices	25.0% decrease (RR∼0.75)	1.0–50.0%	Vissers et al, 2011 [41]

RR: risk ratio; PEP: Post-exposure prophylaxis; STD: sexually transmitted disease; SBIRT: screening, brief intervention and referral for treatment for unhealthy alcohol use; IDU: injection drug use.

1 Values of intervention effect sizes represent relative risk benefits on pathway applied to prioritized population(s) except where noted. For instance, if an intervention included a condom distribution/use component, this would result in a 12.3% increase in the probability of consistent condom usage amongst a specified risk group.

2 The SBIRT intervention acts to reduce the proportion of the population classified as unhealthy alcohol users. The effect size represents the relative decrease in this proportion.

3 The partner services intervention acts to identify previously unknown persons with HIV. The effect size value represents the proportion of undetected HIV positive individuals who move from the “chronic HIV” state to the “in care” state if the intervention is activated.

We sought to identify strategies delivering the greatest health benefit for a particular a budget scenario, also known as efficient frontiers [11]. Strategies outside this frontier are unable to deliver the greatest benefit regardless of budget, and therefore are not preferred choices regardless of available resources. We identified efficient frontiers by calculating the incremental cost-effectiveness ratio (ICER) of combinations of HIV prevention strategies. ICERs measure the additive benefit of each strategy compared with its next best alternative, and interpret this benefit together with its additive cost.

We identified all intervention strategies that the model estimated would be cost-saving and subsequently ran twenty year simulations of each combination of these interventions (n = 16 cost saving interventions, 10 of which were unique; 65,535 possible combinations of any number of n). The intervention portfolios that delivered the greatest benefit for any particular budget scenario (that is, the efficient frontier) were identified using well-established methods [12]. The “optimal” package of interventions we assumed to be represented by the farthest point lying on the frontier as this combination prevented the largest number of infections and yet remained cost-effective to implement.

### HIV transmission

A deterministic compartmental model of HIV transmission was developed, specified by sets of equations. The model was implemented in the C++ programming language. Full details of the conceptualization and parameterization of the model can be found in File S1. The model includes both sexual transmission and transmission through needle-sharing during injection drug use. HIV transmission was modeled using a binomial process and assumed proportionate mixing in the population. The probability of transmission between partners was adjusted to account for infected partner's gender, disease state and treatment status. Differences in risk associated with sexual positioning and positioning preferences between MSM were not considered.

### HIV progression and treatment

Disease progression was modeled by incorporating equilibrium mortality and transition rates between CD4 and HIV-1 viral load (VL) categories as a function of antiretroviral treatment and adherence from a previously described and validated HIV stochastic progression simulation model. Accordingly, the output distributions of the progression model (stochastic) were collapsed into point estimates as a byproduct of enabling the transmission and progression models to exchange information. The progression model explicitly represents the main cause of ART failure, non-adherence leading to the accumulation of genotypic resistance, and has been well-validated in multiple populations [13], [14].

The HIV positive population in the transmission simulation at baseline was divided into compartments based on CD4 and VL strata. Five CD4 strata were represented (<50, 51–200, 201–350, 351–500, >500 cells/mm^3^) and five VL logarithmic strata were represented (<2.5, 2.5–3.5, 3.5–4.5, 4.5–5.5, >5.5 log copies/ml). The spectrum of infection and care was modeled as a stepwise progression from HIV acquisition/primary infection to chronic infection, HIV detection thorough testing, linkage to care, initiation of treatment with antiretroviral therapy, and adherence to therapy.

### Representation of HIV prevention interventions

**Figure 1:**
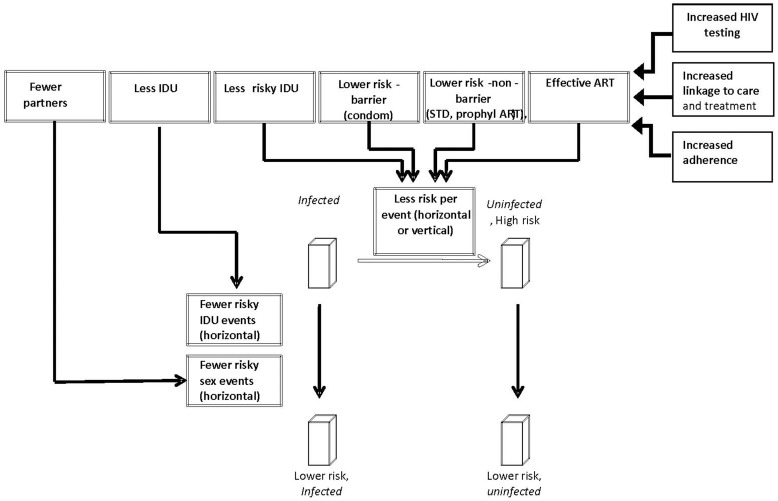
Schematic of constructs in transmission simulation and pathways which impact HIV transmission.

The transmission simulation includes the capacity to represent the implementation of one or more HIV prevention interventions. Each intervention was assumed to impact a specifiable group or population by activating one or more pathways to reduce HIV transmission (see Table 2 and File S1 for further details). Here, ‘pathway’ refers to a fundamental mechanism through which transmission is impacted (e.g. such as reducing the likelihood of unprotected anal intercourse), or by reducing the probability of transmission given that a high-risk act occurs (e.g. such as the likelihood of transmission during unprotected anal intercourse when an HIV positive person is virally suppressed) (Figure 1). Note that while some interventions are restricted to a particular prioritized group by their design (such as medical case management, or care coordination for HIV-positive individuals), other interventions may be applied to multiple alternative groups (for example, a condom distribution intervention can be alternatively directed at HIV-persons with high-risk behaviors, all HIV-infected persons, HIV-negative persons with high-risk behaviors, or all persons). Prioritized groups for a given intervention are represented in the model by specifying particular compartments of specific populations or risk groups (Table 1 and File S1).

The effect of each intervention on each pathway is summarized using the metrics of effect size (Table 2), statistical certitude (95% confidence interval or plausible range) (Table 2), and strength of evidence (“grades” A, B, C, or D), based on the investigator's published adaptation of the evidence rating scale used by the United States Preventive Services Task Force (Table 3) [15]. Any model input with an evidence source that could not be confirmed was conservatively assigned a default grade of “D” (Table 1). An intervention was assigned a single level of evidence equivalent to the “weakest link” in any evidence associated with it (e.g. efficacy of effect on a specific HIV transmission pathway, uptake of intervention) for the purposes of examining effects of filtering by uncertainty.

**Table 3:** Evidence filters for model inputs.

Level of Evidence Filter	Grading Criteria (Assessment of internal validity based on criteria outlined in Braithwaite RS, et al. 2007[15])
A	Systematic review including meta-analysis or individual randomized controlled trial (internal validity: high)
B	High quality observational studies (cohort, case-control; internal validity: high) or lower quality individual randomized controlled trial (internal validity: fair or poor)
C	Lower quality observational studies (internal validity: fair or poor)
D	Expert opinion

Under model scenarios where multiple interventions were incorporated and were hypothesized to affect a similar HIV transmission pathway we assumed conservatively that the effect of the strongest intervention would predominate and the weaker intervention(s) would have no additional effect on that specified pathway (though their effects on any additional unique pathways would be maintained). For instance, if intervention A (acting only through increased condom utilization) prevented 1,000 new infections and intervention B (acting only through increased condom utilization) prevented 2,000 new infections the combination package of A+B would only result in 2,000 new infections being averted. If, however, A or B had additional effects beyond condom utilization the combination of A+B could prevent more than 2,000 infections over the model run.

### Parameter inputs

The population of NYC in 2009 (ages 0–75) based on NYC HIV surveillance data [16] was divided into population compartments based on gender, sexual risk behavior, sexual identity (straight, gay, bisexual), infection status, treatment status, and injection drug use (IDU) (Table 4). Sexual risk was divided into three categories (abstinent, monogamous, multiple concurrent partnerships). Proportions of the population within each sexual risk category remain constant over time. Serial partnerships (i.e. multiple partners in serial fashion over time) were considered as monogamous. Abstinent persons were estimated to represent 21% of the population [17]. The mean CD4 for the initial HIV positive population was 350 cells/mm^3^, and the mean VL was 4.5 log [18]. Persons were defined as “high-risk” (for the purposes of intervention prioritization) if they had multiple sexual partnerships (whether MSM or heterosexual men or women) and/ or were injection drug users (IDUs). Other critical inputs were ascertained from literature estimates, or through discussion and consensus amongst the study team (Table 5). For the purposes of model specification, odds ratios derived from the peer-reviewed literature were converted to appropriate risk ratios using previously defined techniques when necessary [19].

**Table 4:** Initial New York City-based HIV inputs into ECHPP HIV epidemic computer simulation, 2009^1^.

Subgroup	Male HIV+ (known)	Female HIV + (known)	Total HIV+ (known)
Adults (13–65)	76,770	31,596	108,366
*Transmission Risk* 2			
Heterosexual	5,637 (7%)	15,081 (48%)	20,718 (19%)
MSM	35,882 (47%)	–	35,882 (33%)
IDU	15,051 (20%)	6,151 (19%)	21,202 (20%)

1 NYC DOHMH, Bureau of HIV/AIDS Prevention and Control, surveillance data, 2009 [42].

2 Proportion of HIV-positive adults with a reported transmission risk. Proportions do not equal 100% because of persons with unknown transmission risk.

**Table 5:** Generalized inputs into ECHPP HIV epidemic computer simulation.

Parameter or input	Value	Sensitivity analysis limits	Reference
**Sexual risk characteristics**			
Proportion of population who are abstinent	21.0%	17.0–32.0%	Adimora, et al 2007[17]
Probability of monogamous relationship (if sexually active)			
Men who have sex with women (MSW)	78.2%	…	CHS [43]
Men who have sex with men (MSM)	55.8%	…	[43]
Women who have sex with men (WSM)	91.1%	…	[43]
Women who have sex with women (WSW)	48.9%	…	[43]
Probability of multiple partnerships (if sexually active)			
MSW	21.8%	16.1– 23.6%	[43]
MSM	44.2%	25.6–63.6%	[43]
WSM	8.9%	6.9–10.4%	[43]
WSW	51.1%	…	[43]
Proportion of men who are MSM	5.6%	2–10%	[43]
Proportion of men who are MSW	94.4%	…	[43]
Proportion of women who are WSW	2.4%	…	[43]
Proportion of women who are WSM	97.6%	…	[43]
**Injection Drug Use Characteristics**			
Proportion of population that injects drugs	1.43%	1.33–1.91%	Brady JE, et al 2008 [44]
Proportion of injection drug users (IDUs) who have unsafe injection practices	32%	15%–50%	NHBS NYC Data 2009 [45]
Proportion of IDUs who are male	70%	…	NHBS NYC Data 2009 [45]
**Sexual and IDU transmission**			
Transmission risk per sex act			
Male-to-male	0.00167*	…	Jin F [46]; Baggaley[47]
Female-to-male	0.00042	…	Boily[48]
Male-to-female	0.00081	…	Boily[48]
Transmission risk per unsafe needle sharing act	0.003	…	Tokars JL, et al 1993 [49]
Relative risk of transmission if viral load			
0–2.5 log copies/ml	0.16	…	Attia S, et al 2009 [50]
2.5–3.5 log copies/ml	1.87	…	[50]
3.5–4.5 log copies/ml	6.54	…	[50]
4.5–5.5 log copies/ml	8.85	…	[50]
>5.5 log copies/ml	9.03	…	[50]
Sex acts (per partnership) per year	89	69–112	Mosher WD, et al 2005 [51]
Shared injections per year	70	25–100	Assumption
**HIV risk behaviors and biological/behavioral modifiers of transmission**			
Prevalence of untreated sexually transmitted infection	6.9%	0.1–10%	Epiquery—STD registry [52]; Benedetti J, et al 1994 [53]
Prevalence of unhealthy alcohol use	5%	2–10%	Wunsch-Hitzig R, et al 2003 [54]
Prevalence of consistent condom usage	35%	20–50%	CHS [43]
**HIV disease related**			
Probability of annual HIV test	31%	20%–50%	CHS [43]
Probability of linkage to care	75%	…	Unpublished NYC DOMH data
Probability of initiating ART if in care	87%	65–95%	Unpublished NYC DOMH data
**Demographics**			
Age-related mortality rate	0.0068 (6.8/1000 pop)	…	NYC vital statistics, 2009 [16]
Fertility rate	0.0156 (15.6/ 1000 pop/year)	…	NYC Vital statistics 2009 [16]

ART: antiretroviral therapy; * represents an average of different risks per act based on sexual positioning.

Costs were considered from the perspective of the NYC Department of Health and Mental Hygiene (NYC DOHMH) and the City of New York. Costs were derived per intervention from estimates of programmatic expenditures within the DOHMH and did not include costs incurred by other (non-DOHMH funded) agencies. Programmatic costs typically include pro-rated staff time, fixed costs and additional materials required to provide the given intervention (e.g., educational tools or supplies, including the cost of purchasing condoms). Where feasible, fee-for-service rates that incorporate these costs for each unit of service were applied. Plausible cost ranges for many interventions were provided; if no range was given, sensitivity analyses employed ±50% of the estimated cost as the bounds. Annual costs for an intervention were calculated using a “pre-purchasing” perspective – total cost for an intervention equals per unit cost specific to the intervention (cost input from Table 1) times the total number of persons estimated to be in the priority population.

### Calibration, validation, and design features

The base case time horizon is 20 years. We performed sensitivity analyses with alternative time horizons of potential interest for policy decisions (5 years and 10 years). We did not discount costs or benefits. We pre-specified three validation criteria to test whether the model's predictions were compatible with observed results: HIV prevalence, HIV incidence, and HIV-related mortality. We compared data from the most recent year available (2009), as well as time trends over the longest period of time (2003 to 2009) during which NYC data were available for all three criteria. In addition, we tested whether the distribution of new infections across three risk categories (i.e. MSM, heterosexuals and IDU) predicted by the simulation resembles observed results.

## Results

### Model calibration and validation

**Figure 2:**
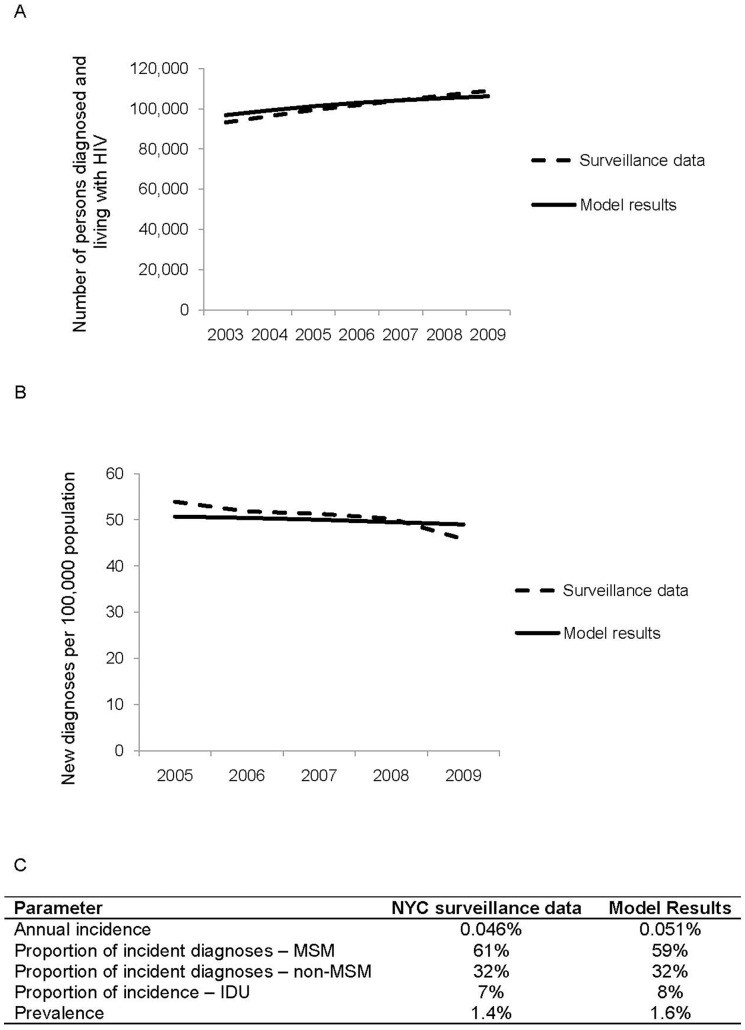
Validation of the HIV epidemic model. **a**. Comparing model prevalence results with reported data for New York City for 2003–2009. **b**. Comparing model incidence results with reported data from New York City 2003–2009. **c**. Comparison of observed versus simulated results, based on most recent year for which DOHMH results are available.

Comparing simulation estimations with epidemiological data from NYC, the simulation demonstrated reasonable goodness of fit with pre-specified validation criteria of HIV incidence and HIV prevalence, over the timeframe for which NYC data was available (2003 through 2009) (Figure 2a–b). In addition, the proportions of new infections among different risk categories (MSM, heterosexuals and IDUs) and the overall prevalence predicted by the simulation during the first year closely resembled the relevant 2009 NYC data (Figure 2).

### Results with baseline investment in HIV prevention programs

Under the base case assumptions, without incremental investment in HIV prevention programs or strategies, the model predicted 58,632 new cases of HIV infection over a 20 year time period, with an average incidence of 2,932 new infections per year. Over the 20 year simulation, 16,159 persons were predicted to have died of AIDS-related conditions, with an average 808 deaths per year.

### Results with increases in investment in HIV prevention programs

Simulation of the implementation of each of the considered HIV prevention interventions resulted in fewer overall number of infections and HIV/AIDS-related deaths than the base case scenario; however, there was notable heterogeneity in the effect and the cost-per-infection averted of each strategy (Table 6). Some of the interventions with the potential to avert the greatest number of new infections (e.g., post-exposure prophylaxis) had a very high cost (>$9 million per-infection averted).

**Table 6:** Selected single policy options, and their impact on HIV infections averted and the cost per infection averted.

Intervention	Target Group	Total cost (x $1million), 20 years	# new infections, 20 years	# infections averted, 20 years	Cost per Infection Averted	Favorable Value (Yes/No)
Base case (No additional interventions)	N/A	N/A	58,632	N/A	N/A	
Condom distribution	HIV-infected, high-risk	$4.5	57,118 (58,227–55,977)	1,514 (405–2,655)	$2,969 ($1,690–11,100)	Yes
Social marketing	HIV-infected	$18.6	53,280 (48,287–57,895)	5,352 (737–10,345)	$3,474 ($1,770–25,500)	Yes
Condom distribution	HIV-infected	$13.5	56,321 (54,581–58,014)	2,312 (619–4,052)	$5,854 ($3,326–21,966)	Yes
Community intervention	All	$82.9	47,071 (37,701–57,085)	11,562 (1,548–20,931)	$7,173 ($3,962–53,570)	Yes
Prioritized use of surveillance data	HIV-infected	$16.7	58,029 (57,480–58,560)	603 (73–1,152)	$27,663 ($14,405–230,854)	Yes
Cofactors	HIV-infected, high-risk.	$65.5	56,540 (55,744–57,344)	2,092 (1,288–2,888)	$31,304 ($25,948–58,741)	Yes
SBIRT	HIV-infected, hazardous alcohol users	$11.6	58,316 (58,250–58,381)	317 (251–382)	$36,772 ($35,032–53,330)	Yes
Social marketing	Providers	$715.6	49,832 (42,565–57,467)	8,801 (1,165–16,607)	$81,315 ($44,544–614,177)	Yes
Social marketing	All	$954.2	47,071 (37,701–57,085)	11,562 (1,548–20,931)	$82,532 ($45,595–616,407)	Yes
Linkage to care	HIV-infected	$59.6	57,852 (56,860–58,426)	780 (206–1,772)	$380,906 ($161,007–1,564,241)	Yes
Social marketing	HIV-uninfected, high-risk	$935.1	49,997 (43,377–57,203)	8,635 (1,429–15,255)	$108,291 ($61,314–654,404)	Yes
Condom distribution	HIV-uninfected, high-risk	$358.5	55,847 (53,771–57,884)	2,785 (748–4,861)	$128,715 ($73,747–479,120)	Yes
Linkage to support	HIV-infected	$1,681.9	45,100 (37,198–54,584)	13,532 (4,048–21,434	$124,291 ($76,929–425,376)	Yes
Condom distribution	All	$590.3	55,479 (53,132–57,785)	3,153 (847–5,501)	$187,212 ($107,311–696,563)	Yes
Partner services	HIV-infected and partners	$74.0	58,259 (58,232–58,288)	373 (344–400)	$198,253 ($184,854–215,195)	Yes
STD screening	HIV-infected, high-risk	$332.1	57,653 (57,380–57,966)	980 (666–1,253)	$339,026 ($264,888–499,101)	Yes
STD screening	HIV-infected	$501.1	57,584 (57,291–57,919)	1,048 (713–1,341)	$477,984 ($373,509–703,563)	No
Risk reduction	HIV-infected	$4,107.7	53,280 (48,287–57,895)	5,352 (737–10,345)	$767,431 ($391,903–5,637,789)	No
Social services	HIV-uninfected, high-risk	$3,986.6	54,822 (51,710–58,082)	3,810 (550–6,922)	$1,046,387 $568,274–7,340,070)	No
Care coordination	HIV-infected, on ART	$12,597.5	47,755 (41,841–54,717)	10,877 (3,915–16,791)	$1,158,199 $740,254–3,268,504)	No
Testing – clinical	HIV uninfected	$8,124.0	54,024 (52,036–55,831)	4,608 (2,801–6,597)	$1,763,061 ($1,231,602–2,899,854)	No
Testing – non-clinical	HIV-uninfected	$13,110.1	54,417 (51,444–57,566)	4,215 (1,066–7,188)	$3,110,381 ($1,823,909–12,298,571)	No
Cofactors	HIV-uninfected, high-risk	$2,298.8	57,999 (57,592–58,407)	633 (225–1,040)	$3,631,257 ($2,537,148–11,767,147)	No
SBIRT	HIV-uninfected, high-risk	$540.9	58,493 (58,442–58,544)	139 (88–190)	$3,895,458 ($3,276,457–7,079,913)	No
PEP HR(-)	HIV-uninfected, high-risk	$176,466.0	40,632 (41,427–52,469)	18,000 (6,164–17,205)	$9,803,449 ($10,256,032–28,602,672)	No
STD screening HR(-)	HIV-uninfected, high-risk	$15,437.6	57,279 (56,903–57,711)	1,354 (921–1,730)	$11,404,509 ($8,924,995–16,758,381)	No
PEP	HIV-uninfected	$284,790.0	39,042 (26,554–46,910)	19,590 (11,722–32,076)	$14,537,519 ($8,884,247–24,285,093)	No
STD screening – all	All	$25,423.1	57,191 (56,791–57,651)	1,441 (981–1,841)	$17,640,475 ($13,805,927–25,920,175)	No

Results are shown for infections averted over a time horizon of 20 years. Costs reflect *additional increases* in expenditures. An intervention is considered to be of favorable value if cost-per-infection averted <$360,000). Values in parenthesis represent upper and lower bounds of estimates related to assumptions regarding intervention efficacy (lower, upper).

SBIRT: screening, brief intervention and referral for treatment for unhealthy alcohol use; STD: sexually transmitted disease; PEP: Post-exposure prophylaxis.

HR(−): high risk, HIV-uninfected.

### Analysis limited to cost-saving interventions

A group of ten unique interventions had the potential to be cost-saving: condom distribution; social marketing; community-based prevention; prioritized use of surveillance data (i.e., targeted use of HIV and STD surveillance data to prioritize risk reduction counseling and partner services for persons with previously diagnosed HIV infection with a new STD); cofactor risk reduction; screening, brief intervention and referral for treatment for unhealthy alcohol use (SBIRT); linkage to care; linkage to support services for HIV-positive persons; partner services (defined here as just partner notification and testing); and STD screening. These ten unique interventions could avert each new HIV infection at a lower cost than the estimated downstream cost of that infection [10].

**Figure 3:**
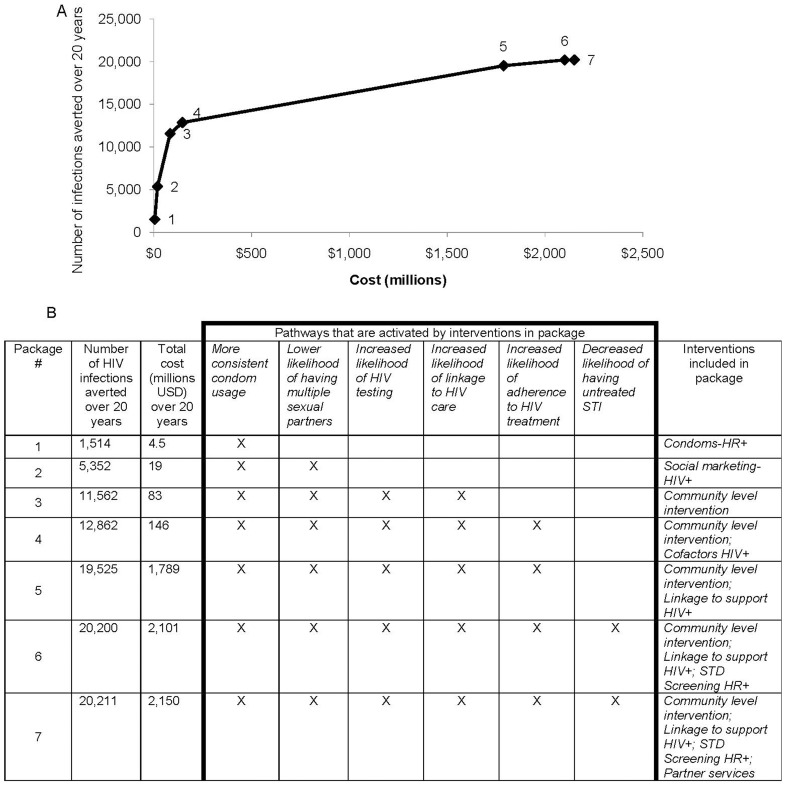
Efficient frontier for HIV prevention interventions found to have “favorable value” during a 20 year simulation of HIV epidemic in NYC. **a**. Graphical representation of frontier. *Diamonds represent packages of intervention(s) on the frontier*. **b**. Interventions and the pathways they activate contained within each efficient frontier package. *X, pathway activated within package*.

When the simulation evaluated all possible combinations of these ten interventions (16 non-unique interventions) that are potentially cost-saving and sought to identify the package of interventions that would avert the most HIV infections for particular budget scenarios, seven of these interventions were included in the different packages located on the efficient frontier, including condom interventions (prioritized for high-risk HIV-infected persons), social marketing for HIV-infected persons, community interventions, interventions to address cofactors for HIV-infected persons, linkage to support for HIV-infected persons, and partner services (Figure 3). For an additional budget of <$1 million USD annually, a social marketing campaign focused on persons living with HIV could avert an additional 5,352 (9%) new HIV infections over the next twenty years (Package 2; Figure 3).

**Figure 4:**
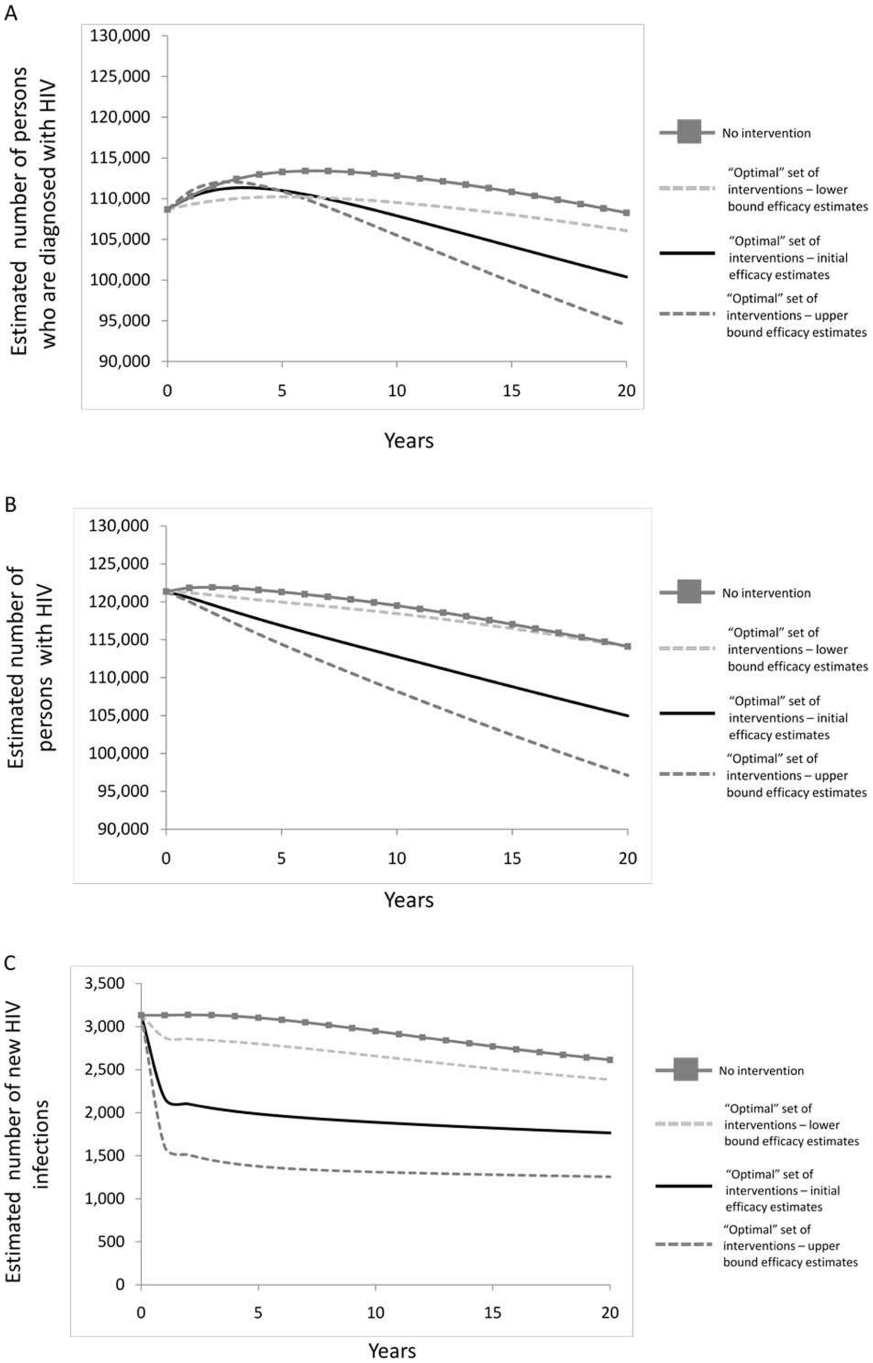
Epidemic curves for 20 year simulation. *Black line*- Base case scenario; *Grey line*- optimized package (Package 7 from Figure 3b) implemented. **a**. New HIV diagnoses over 20 years. **b**. HIV prevalence over 20 years. **c**. HIV incidence over 20 years.

The package of potentially cost-saving interventions predicted by the model to prevent the most infections was implementation of evidence-based community-level interventions, STD screening for high-risk HIV infected persons, partner services, and a linkage to support interventions (Package 7; Figure 4). Such a package would result in 20,211 (34%) of new HIV infections averted. The cost per infection averted for this package is predicted to be $106,378; however, the total cost savings would be more than $5 billion (or approximately $250 million per year, on average) because the $2 billion of program costs over the 20 year time horizon would be offset by the predicted downstream savings from infections averted totaling more than $7 billion. This package would result in a corresponding early increase in prevalence followed by a later decline, reflecting the package's impact on the kinetics of detection and entry into care (Figure 4).

### Analyses considering all interventions regardless of cost

The package of interventions predicted by the model to prevent the greatest number of infections (without regard for cost, in order of strongest effect) included expanded provision of post-exposure prophylaxis for HIV uninfected persons, linkage to support, social marketing for HIV-infected persons, evidence-based community level interventions, and enhanced HIV testing in clinical settings. An estimated 33,004 (56%) of infections would be averted implementing this package of interventions at an estimated cost-per infection averted of nearly $9 million (see File S2).

Implementation of a package of interventions representing a “test and treat” only strategy (i.e., enhanced HIV testing in clinical settings, linkage to care intervention and care coordination intervention) without some of the other interventions listed above included in the portfolio, assuming uptake of testing, linkage and treatment at levels predicted in the literature, resulted in 14,048 (25%) infections averted during the twenty year simulation. However, near perfect efficacy of “test and treat” (i.e., universal annual screening, immediate linkage to care, universal ART, and perfect adherence to ART) predicted that >80% of new HIV infections would be averted and the cost-per-infection averted would be <$360,000. In addition, reduction by a factor of ten in the cost of the “test and treat” package rendered the intervention cost-saving even under the base case efficacy assumptions.

### Sensitivity analysis

Several of the interventions had >10% absolute change in their projected effectiveness in one-way sensitivity analysis (see File S2). Varying all parameters (listed in Tables 1–2 and 4–5) across the plausible ranges for each and evaluating the effects of all interventions under these conditions demonstrated that the prevention interventions considered to be of favorable value were robust. No intervention with a cost-per-infection averted greater than the $360,000 threshold under base case assumptions crossed this threshold under any other conditions. However, several of the interventions, including condom distribution to high risk, HIV-negative persons; linkage to support; condom distribution to the whole population; partner services; and STD screening for high-risk, HIV-infected persons, that were considered cost-saving under base case assumptions had cost-per-infection ratios which increased above the threshold considered as cost-saving under other, specific conditions (see File S2).

Under conditions where ART initiation was not restricted by CD4 count (as has been recommended by the DHHS [20]) there were no differences in the list of interventions that were considered to be cost-saving or in the relative rankings of interventions by cost-per-infections averted (data not shown). Similarly, when we varied the time horizon of the analysis, the group of interventions considered cost-saving did not change (see File S2).

### Effects of optimization by level of evidence

The efficient frontiers of combined HIV prevention interventions were highly dependent on quality of evidence criteria. If analysis was limited to only those packages supported by the strongest evidence (Level A), only condom distribution interventions would be included in the intervention portfolio. However, as evidence limitations were relaxed to include all interventions supported by at least some observational data, more interventions were included in the intervention portfolio, and more infections could be averted, albeit at a higher cost (see File S2).

## Discussion

We have developed an innovative, jurisdiction-specific simulation that can identify the most cost-effective portfolio of interventions to maximize HIV infections averted in a major urban area of the United States for a defined budget. Great variation was found in the cost per infection averted by the HIV prevention strategies considered, ranging more than 1,000-fold. Ten of these interventions prevented new HIV infections at favorable value, with costs-per-infection averted falling below the expected downstream costs of the HIV infections (had they occurred). Our results suggest that the highest value interventions focus on individuals already HIV-infected, rather than the much larger number of individuals who are not known to be HIV-infected, reinforcing conclusions from Lasry et al [21]. While not suggesting that prevention resources should be targeted exclusively to HIV-infected persons, our results do indicate that altering the balance of services in favor of HIV-infected persons, particularly those at high risk of onward transmission, may avert a high proportion of new infections at relatively low cost.

While the results varied somewhat in sensitivity analyses that considered statistical uncertainty, they did not vary sufficiently to alter likely decisions about intervention prioritization. A similar group of interventions fell below the cost-of-infection threshold, regardless of the optimism of assumptions or the time horizon used. Results do appear to be very sensitive to analyses that considered uncertainty of level of evidence. When we required that only level A and B evidence interventions were considered, the optimized package of interventions could only avert 6% of infections as compared to 23% when relaxing the evidence criteria to include all intervention strategies supported by some observational data (levels A–C).

While others have reported results of a model using an extremely optimistic “test and treat” strategy in the South African context, suggesting potential reductions in HIV infections of up to 95% [4], our model found much more conservative results, albeit with much more conservative assumptions. With base-case assumptions, an “optimized” package of non-ART dependent interventions would reduce new infections by 20–30%, whereas under assumed thresholds a “test and treat” strategy alone would reduce new infections to a lesser degree (∼25% reduction), but at a greater cost. However, if we are able to approach the theoretical limits of “test and treat” efficacy (universal annual screening, immediate linkage, universal ART and perfect adherence) near elimination of ongoing HIV transmission could be realized. Our results are consistent with model based cost-effectiveness estimates of similar intervention strategies published previously [7], [8], [21], [22].

For the many evidence-based interventions that can prevent large numbers of infections but only at very high costs per infection averted (e.g., PEP, adherence interventions) scale up across a large segment of the population who may be at low risk of HIV acquisition or transmission may be cost prohibitive. For those individuals with both high infectivity and ongoing behavioral risk (for example, a probability of infecting at least one other person of greater than 10% per year), up to $18,000 could be spent per year (over 20 years) on a highly tailored package of HIV-reduction interventions for that person while still spending less than $360,000 to avert each infection. Our results suggest the potential benefit of developing even more sophisticated operations research to prioritize the allocation of resources to these individuals more effectively, despite the inherent challenges of doing so.

It is important to note that NYC's high rates of testing (31% of adult NYC residents reported HIV testing in past 12 months in 2009) and linkage to care (75% of persons diagnosed were linked to care in 3 months in 2009 using prevailing definitions) may have had an important impact on our findings because we analyzed marginal rather than absolute resource allocation questions (i.e., additional benefit from increased funding rather than expected benefit from existing funding). Interventions to improve linkage to care had comparatively small effects, and correspondingly unfavorable cost-effectiveness, because the vast majority of newly infected NYC were already linked during the period of study. Similarly, interventions to improve testing rates had comparatively small effects, and correspondingly unfavorable cost-effectiveness, because a substantial proportion of New Yorkers were already tested for HIV annually during the period of study.

### Limitations

Like any computer simulation, not all inputs are known with certainty, and results are partially dependent on the assumptions embedded in the model. Costs in our model were not addressed from the comprehensive societal perspective, but were rather assessed based primarily on the costs to local public health authorities in NYC. They may not be inclusive or reflective of all costs incurred by society or specific payers. Specifically, the cost of antiretroviral therapy for treatment is not included here, largely because this particular model sought to specifically address the list of initial CDC ECHPP interventions on which this model was originally based. A related limitation is that some of the more innovative and recently approved biomedical interventions, such as rapid HIV self-testing and pre-exposure prophylaxis (neither of which were part of ECHPP nor FDA-approved at the time of model development/validation) were not modeled here. Modalities still under investigation, such as microbicides or HIV vaccines, were also not included. Further, interventions considered in the model are not always discrete (i.e., interventions may impact more than one of the components/pathways), and some may be defined more narrowly in the model than they are implemented in reality (e.g., partner services can link persons to care and services and distribute condoms in addition to partner notification and testing). In general, interactions between different interventions are also not taken into account here, either wasteful or synergistic.

Per-person costs in our model were derived from programmatic estimates from the DOHMH and were applied in a “pre-purchased” approach (i.e., extrapolating the cost to assume that every intervention is purchased every year in a sufficient quantity to reach every person in the target population). This neither accounts for the potential economies of scale that may be operational nor the actual utilization of an intervention (as represented by its effect size in our model). Therefore, potential bias towards overestimation of costs of interventions may occur, leading to a more conservative estimate of portfolios of interventions that may be “cost-saving.” This bias may explain, in part, why expanded HIV testing in our model appears to be less cost-effective than it was found to be in other published mathematical models [23], [24]. In addition, our model does not explicitly consider costs of the antiretroviral medications or the routine care needed by a person living with HIV/AIDS, although these costs informed the estimation of the $360,000 threshold.

Assumptions we made may have also contributed to the model's limitations. We made assumptions about the mechanisms of action of the HIV prevention interventions and the lack of interactions between interventions when more than one was implemented as a part of a specific package. Our mapping process (assessing which pathways were influenced by which interventions) was reviewed and agreed upon by members of both the academic modeling team and the DOHMH, and many of these assumptions were based on expert opinion where sufficient data was unavailable or inadequate. There are little to no reliable data to inform how different interventions would impact on each other if implemented in tandem. We chose a conservative approach by hypothesizing that specific interventions act through mutually exclusive mechanisms and that a pathway for a specific person/population could only be “activated” once no matter how many interventions affected it.

### Conclusions

This computer simulation, constructed using operations research methods, may be useful to inform program and policy decisions for HIV prevention and care in NYC and other major urban areas. Based on the needs and settings of particular decision-makers, this model can represent the interplay between different combinations of interventions and can generate highly jurisdiction-specific results. After validation of this model using inputs from the NYC epidemic and incorporating the interventions prioritized in CDC's ECHHP project, these results suggest that many infections can be prevented at acceptable cost by systematically prioritizing and implementing known interventions. Preliminary results from this modeling effort were used, in part, to help inform the development of a new solicitation for HIV prevention services in New York City as well as pilot clinic-based activities that prioritize secondary HIV prevention interventions among persons living with HIV in NYC.

## Supporting Information

File S1 **Supporting text, tables, and figures. Supporting Methods. Table** **S1.** Components of population matrix. **Figure** **S1.** Schematic diagram of HIV transmission model. **Figure** **S2.** Who Can Partner With Who matrix depicting possibility of a partnership occurring between two groups (defined by gender and sexual orientation). **Table** **S2.** Input parameters. **Figure** **S3**. Probability distributions for initialization of CD4 count strata and VL strata within HIV infected compartments. **a**. Probability distribution (Q′*_cd,v_*) of CD4 and VL categories for HIV infected persons on ART at initialization. **b**. Density map of probability distribution Q′. **c**. Probability distribution (Q*_cd,v_*) of CD4 and VL categories for HIV infected persons not on treatment at initialization. **d**. Density map of probability distribution Q*_cd,v._* **Figure** **S4**. HIV transmission “pathways” that are influenced by prevention interventions. **a**. Schematic of constructs in transmission simulation and pathways which impact HIV transmission. **b**. Pathway mechanisms. **Table** **S3**. ECHPP interventions, HIV transmission pathway mapping and targeted populations. **Table** **S4**. New York City derived HIV data and transmission risks in 2009. **Table** **S5**. Calculated initial population distribution across risk strata and HIV infection spectrum of care/engagement.
(DOCX)

File S2 **Supporting text, tables, and figures. Additional results. Figure** **S5.** *Efficient frontier of most efficacious packages of HIV prevention strategies in NYC over 20 years*. **a**. Packages (1–7) consist of those combinations of the 16 most effective (as measured by # of infections averted) interventions that have the most favorable incremental cost to effectiveness ratios. All other combinations of the 16 considered interventions fall to the right of the curve and are therefore not preferred. **b**. Table which provides details on the 7 packages which lie on the efficient frontier including the specific pathways activated by the package of interventions. **Figure** **S6**. *One way sensitivity analyses for effectiveness and cost-effectiveness ratio of HIV prevention strategies in NYC*. **a**. Range of values for % of HIV averted for each intervention when all input parameters are varied across their spectrum of values that were considered. **b**. range of values for cost-per-infection averted among those interventions found to be cost-saving under reference case assumptions. *Note that STD screening intervention effect size plausible range includes a null effect, therefore, the upper limit of the cost-per-infection averted parameter for this intervention is undefined.* **Table** **S6**. Alternate time horizons (5, 10 years) of computer simulation and comparative effectiveness of HIV prevention strategies in NYC. **Figure** **S7**. *Effects of optimization by level of evidence*. **a**. Efficient frontier of combinations of HIV prevention strategies filtered by level of evidence. Packages represent combinations of only those strategies that met or exceeded a specified level of evidence and which had an ICER that was of optimal value. All other combinations fall to the right of the curve and are therefore not preferred (and not shown on the figure). Level A (denoted by a purple triangle) included only those interventions with an evidence grade of A; Level B (denoted by a green square) included interventions with level of evidence grade A or B; Level C (denoted by a black diamond) included interventions with level of evidence grade A or B or C. No optimization curve could be generated for all interventions (i.e. any evidence grade) because of a limitation of computing resources and runtime necessary. **b**. Table which provides details on the packages which lie on the efficient frontier including the specific pathways activated by each package of interventions.
(DOCX)
