# Supplementary material for: Averting HIV Infections in New York City: A Modeling Approach Estimating the Future Impact of Additional Behavioral and Biomedical HIV Prevention Strategies
Source: PLoS One. 2013 Sep 13;8(9):e73269. doi: 10.1371/journal.pone.0073269 (PMC3772866; doi:10.1371/journal.pone.0073269)
Supplement: File S1 — Supporting text, tables, and figures. Supporting Methods. Table S1. Components of population matrix. Figure S1. Schematic diagram of HIV transmission model. Figure S2. Who Can Partner With Who matrix depicting possibility of a partnership occurring between two groups (defined by gender and sexual orientation). Table S2. Input parameters. Figure S3. Probability distributions for initialization of CD4 count strata and VL strata within HIV infected compartments. a. Probability distribution (Q′cd,v) of CD4 and VL categories for HIV infected persons on ART at initialization. b. Density map of probability distribution Q′. c. Probability distribution (Qcd,v) of CD4 and VL categories for HIV infected persons not on treatment at initialization. d. Density map of probability distribution Qcd,v. Figure S4. HIV transmission “pathways” that are influenced by prevention interventions. a. Schematic of constructs in transmission simulation and pathways which impact HIV transmission. b. Pathway mechanisms. Table S3. ECHPP interventions, HIV transmission pathway mapping and targeted populations. Table S4. New York City derived HIV data and transmission risks in 2009. Table S5. Calculated initial population distribution across risk strata and HIV infection spectrum of care/engagement. (DOCX) [file pone.0073269.s001.docx]

Supplementary Materials S1:

Methods

1. **HIV Epidemic Model**

We developed a model to represent the interplay between transmission via heterosexual contact, homosexual contact, needle-sharing behaviors and a variety of HIV prevention activities. This model incorporates equilibrium results from a previously validated stochastic model of HIV disease progression with a new deterministic compartmental model [[1](#_ENREF_1)]. We sought to evaluate how additional interventions/resource expenditures would impact the HIV epidemic in a complex urban environment. The base case scenario represents the current state of HIV prevention expenditure and efficacy which is not modeled explicitly, but accounted for in the calibration and validation of the model.

For additional information or for a working copy of the C++ code used to conduct the analyses outlined in this manuscript please contact the lead of our mathematical modeling team at Kimberly.Nucifora@nyumc.org.

- 1. **Population definition**

The population of New York City was divided into two age groups (*a*), two genders (*k*), three sexual orientations (*i*), three sexual activity classes (*l*), and two intravenous drug use classes (*j*). These risk groups were selected to capture differences in HIV prevalence, population size, sexual and drug use behaviors and transmission risk. For those persons living with HIV/AIDS their disease was represented with a CD4 count category (*c*) and a VL category (*v*). In addition, the spectrum of infection and engagement in care was represented in a status category (*y*) (Table S1). The specific combination of gender, orientation, sexual activity class and IDU may be (where appropriate) referred to using “risk strata” (subscript *r)* throughout the remainder of this document.

**Table S1.** Components of population matrix

| **Population parameter** | **Abbreviation/Subscript** |  | **Subgroups** |
| --- | --- | --- | --- |
| Age | *a* |  | Children*(a=1),* Adult *(a=2)* |
| Gender | *k* | *r* | Women(*k=1*), Men (*k=2*) |
| Sexual orientation | *i* |  | Heterosexual *(i=1)*, Homosexual *(i=2*), Bisexual *(i=3)* |
| Sexual activity | *l* |  | Abstinent (*l=1*), Monogamous (*l=2*), Multiple partnerships (*l=3*) |
| Intravenous drug use | *j* |  | IDU (*j=1*), non-IDU (*j=2*) |
| CD4 count | *cd* |  | 0-50, 51-200, 201-350, 351-500, >500 cells/mm^3^ |
| VL | *v* |  | 0-2.5, 2.5-3.5, 3.5-4.5, 4.5-5.5, >5.5 log copies/ml |
| Status | *y* |  | Susceptible (y=1), Acute HIV infection (y=2), Chronic HIV infection (not in care) (y=3), Chronic HIV (in care) (y=4),  Chronic HIV (on treatment) (y=5) |

- 1. **Epidemic Compartmental Model**

The compartmental model of HIV transmission, progression and mortality was created through a system of nonlinear differential equations for each spectrum of care group (referred to as status in Table S1) further subdivided by age (a), CD4 category (cd), VL category (v), and risk group (r). Across the spectrum of care, transition from susceptible (S_r_) to acute HIV infection (I^1^_r_) occurs as a result of new transmission events; transition from (I^1^_r_ ) to undetected, chronic HIV infection (I^2^_r_ ) occurs at a constant rate and represents the natural history of acute HIV infection; transition from I^2^_r_ to HIV, in care (I^3^_r_) occurs as a result of HIV testing and linkage to care and this rate can vary in relation to changes in these probabilities. Finally, transition from I^3^_r_ to HIV, on ART (I^4^_r_) occurred once those in care initiated ART. From I^4^_r_ the only transition that could be made was to death. [Figure S1] As discussed below, HIV progression (i.e. transitions between CD4 and VL compartments) and HIV-related mortality was modeled using rates developed from a stochastic state-transition model [[1](#_ENREF_1)].

**Figure S1.** Schematic diagram of HIV transmission model


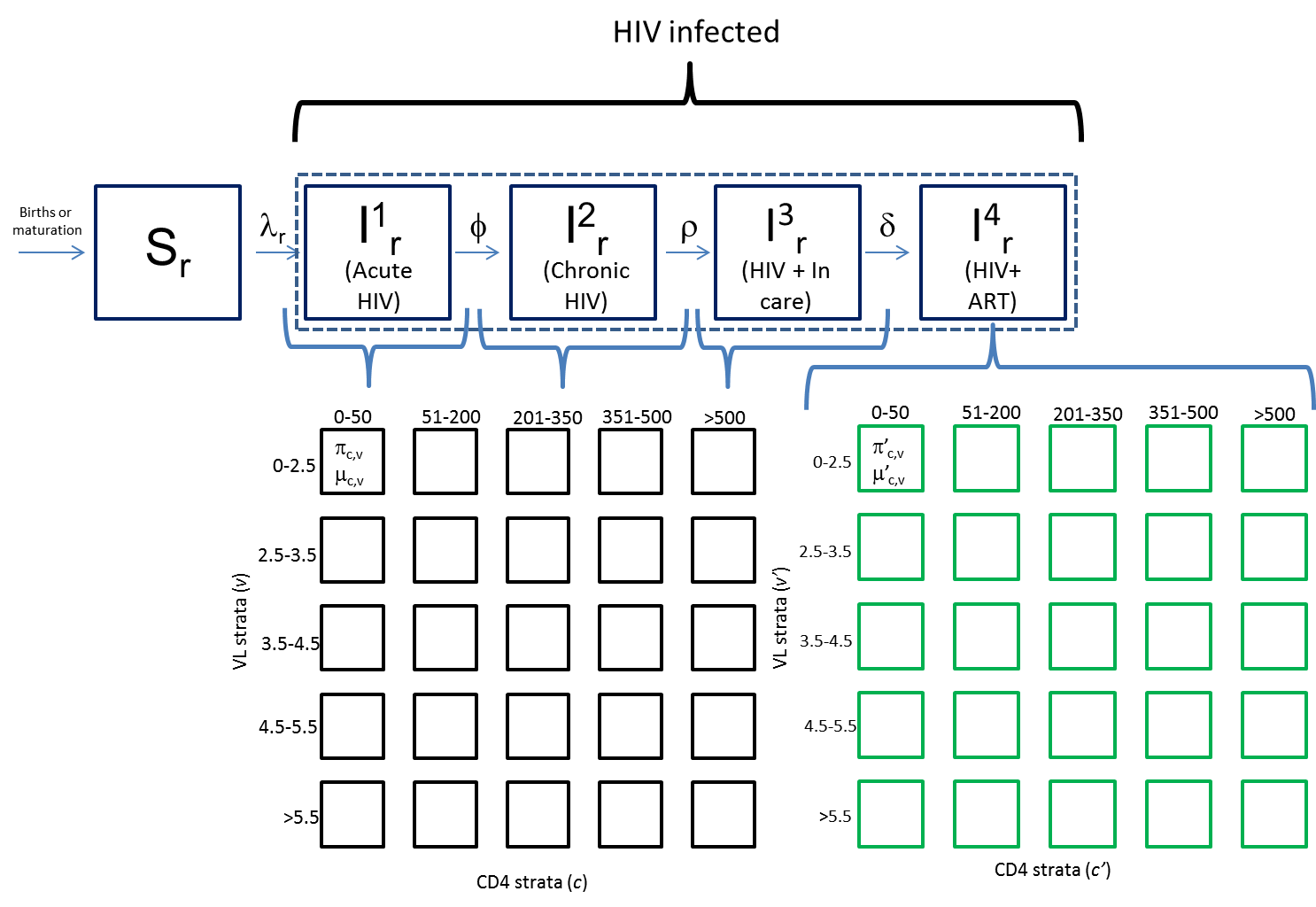


HIV transmission interface

HIV progression model interface

- 1. **Equations governing transitions**

N = Total population

N’ = HIV infected population

S = N-N’ = Susceptible (HIV-) population

r = risk strata- given combination of gender (k), sexual orientation (i), IDU (j), sexual activity class (l)

μ_cd,v_ = HIV-related mortality rate (for a given CD4 strata (cd), VL strata (v))

μ’_cd,v_ = HIV-related mortality rate if on cART

μ_age_ = age – related mortality rate

λ = force of infection

γ = birth or maturation

π = rate of transition between any two CD4, VL strata

φ = flow from acute to chronic HIV infection

η = rate of transition from acute to chronic HIV infection

ν = rate of detection and linkage to care

δ = rate of initiation of ART (once in care)

P_test_= probability/rate of annual HIV testing

P_LTC_= probability/rate of linkage to care (if detected)

P_ART_= probability/rate of initiation of ART (if in care and CD4<500)

1. *d*S_r_/*d*t _=_ γ _r_ – λ^t^_r_ (*t*)S_r_ – μ_age_
2. *d*IHVL_r_/*d*t = λ^t^_r_ (*t*)S_r_ – φ_r_ - μ_age_ - μ*_cd,v_* (where φ_r_ = η*N’_r_)
3. *d*ICHR_r_  / *d*t = φ_r_ - ν_r_ - μ_age_ - μ*_cd,v_* (where ν_r_ = (PLTC)(Ptest))
4. *d*ICARE_r_/*d*t = ρ_r_ – δ_r_ - μ_age_ - μ*_cd,v_* (where δ_r_ = N’_r_*π*_cd<threshold,v_* * PART)
5. *d*IART/*d*t = δ_r_ - μ_age_ - μ’*_cd,v_*
   1. **HIV transmission**

The model captures transmission via heterosexual contact, homosexual contact and needle-sharing practices. Sexual and IDU transmission modes were modeled independently. For the nomenclature used to characterize the model processes below lower-case subscripts refer to the population/compartment for which the calculated value is being performed while the upper case subscripts refer to the partner characteristics.

1.4.1 Sexual Transmission

The population is described by two genders (male and female), three orientations (exclusive homosexual, exclusive heterosexual, and bisexual) and three activity groups (abstinent, low, high). For each sexual activity risk group we assumed one value for the number of new sexual partners a person in that risk group would have annually (partner change rate).

The possibility of a partnership occurring between two groups is described by the Who Can Partner With Who matrix (W), as follows (Figure S2). A heterosexual person can partner with a heterosexual or bisexual person of the opposite sex. A homosexual person can partner with a homosexual or bisexual person of the same sex. A bisexual person can partner with a homosexual or bisexual person of the same sex or a heterosexual or bisexual person of the opposite sex.

Thus, the Who Can Partner With Who matrix (W) is described by a binary value (1 for possible (green), 0 for not (red)) for any person of gender (k) and orientation (i) with a partner of gender (K) and orientation (I), written W_kKiI_, as follows:

**Figure S2.** Who Can Partner With Who matrix depicting possibility of a partnership occurring between two groups (defined by gender and sexual orientation)

|  | | **I can partner with…** | | | | | |
| --- | --- | --- | --- | --- | --- | --- | --- |
|  |  | Male, heterosexual | Male , homosexual | Male , bi | Female, heterosexual | Female, homosexual | Female, bi |
| **I am a…** | Male , heterosexual | 0 | 0 | 0 | 1 | 0 | 1 |
|  | Male , homosexual | 0 | 1 | 1 | 0 | 0 | 0 |
|  | Male,  bi | 1 | 1 | 1 | 1 | 0 | 1 |
|  | Female,  Heterosexual | 1 | 0 | 1 | 0 | 0 | 0 |
|  | Female, homosexual | 0 | 0 | 0 | 0 | 1 | 1 |
|  | Female, bi | 1 | 0 | 1 | 1 | 1 | 1 |

A level of assortativeness (ε) is used to describe the partnering pattern between activity classes where 0 represents the fully assortative case and 1 the fully proportionate case. The mixing and balancing of sexual partnerships is based on previously published work.[[2](#_ENREF_2)]

The proportionate mixing matrix ρ^p^ is defined by:

ρ^p^_kKiIlL_ = N_KIL_c_L_W_kKiI_ / ΣΣΣN_XYZ_c_Z_W_kKiI_ (1)

The term ρ*_kKiIlL_* is the conditional probability that the sex partner of someone of

sex *k,* sexual activity class *l*, and orientation *i* is of sex K, in sexual activity class L and has an orientation I.

The value *c_L_* is the mean rate at which someone in sexual activity class *l* acquires new sexual partners. In equation (1) X= all possible genders, Y = all possible orientations, Z= all possible sexual activity classes.

If ρ^a^_kKiIlL_ represents the fully assortative case

The fully assortative mixing matrix ρ^a^ is defined by:

ρ^a^_kKiIlL_ = N_KIL_c_L_W_κKιI_δ_lL_/ ΣΣΣN_XYZ_c_Z_W_kKlL_δ_lL_ (2)

Here δ_lL_ is the Kronecker delta (i.e. δ_lL_ = 1 if l = L and 0 if not).

The overall mixing matrix based on the desired level of assortativeness ε is:

ρ_kKiIlL_ = ερ^p^_kKiIlL_ + (1 – ε)ρ^a^_kKiIlL_ (3)

The force of infection resulting from sexual transmission is the rate per year at which susceptibles acquire infection. This rate, **λ^s^** , is defined by

λ^s^*_kil_* (t) = ΣΣΣ (c_L_ ρ _kKiIlL_ * ΣΣ β^s^_kKV_ Y_KILV_(t) / N_KIL_(t)) (4)

The transmission probability β^s^*_kKV_* is defined per partnership from someone of sex *K* in a viral load strata V to a susceptible in gender class k.

- - 1. Calculation of probability of transmission per act and per partnership

We refer to the transmission probability per partnership as β and the transmission probability per sexual act as α. Alpha constants were set based on literature reviews and vary by type of partnership and the infecting partner (Table S2). These constants can be modified by HIV disease status (acute vs chronic infection), viral load, adherence and specific interventions which act or promote different behavioral or biological modifiers that increase or decrease this probability (e.g. condom use during sex act will decrease the probability of transmission).

Therefore, under different conditions α_kKV_ is calculated using the following equations:

*Infected partner not receiving treatment (and no alpha modifying interventions):*

α_kKV_ = α_kK_ * α^m^_v_ (5)

where α^m^_v_ is the alpha modifier for a viral load strata *v*

To account for the higher VL (and likely transmission probability resulting) associated with the acute HIV state, if the partner of a susceptible person was in an acute HIV state (I^1^_r_) then the viral load of the infected person was conservatively estimated to be 1.5 log units greater than its set point (and a different VL modifier (α^m^_v_) applied to the above equation [[3](#_ENREF_3)].

*Infected partner receiving treatment (and no alpha modifying interventions):*

First, an “on treatment” viral load strata for infected population is determined as follows:

VL_treat_ = VL - (VLdec * P_adh_)

and then

VL_treat_ is then assigned a new VL strata (*v’ )* category

α^mt^_v_  is the alpha modifier of the new VL strata on treatment *v’*

The final alpha value is then calculated as in equation (5).

For conditions where specific interventions are implemented see section on Interventions below.

To calculate β we assumed a binomial process where the number of trials referred to the avg number of sex acts per partnership (APP) and the probability of successful transmission is described by α_kKV_ as above. Therefore, beta was calculated using the following equation.

β_kKV_ = 1 -(1 - α_kKV_ )^APP^ (6)

1.4.3 Transmission via unsafe needle sharing

There is no mixing matrix to describe the sharing of needles between IDUs, therefore, the probability of needle sharing between risk groups only relates to their relative proportion in the population. Therefore, the force of infection for transmission via needle sharing is described by the following equation:

λ^I^ = c_IDU_β^I^_V_ Y_IV_(t) / N_IV_(t) (7)

Where c_IDU_ refers to the needle sharing partner exchange rate and β^I^_V_ refers to the transmission probability of HIV from an infected person with viral load strata V to a susceptible IDU.

- - - 1. Calculation of probability of transmission per needle sharing act and per partnership

For transmission resulting from needle sharing the probability of transmission was set based on literature review (see Table S2). This probability can be modified by viral load, and adherence to ART.

Therefore, under different conditions α^I^_V_ is calculated using the following equations:

*Infected partner not receiving treatment (and no alpha modifying interventions):*

α^I^_V_ = α^I^_V_ * α^m^_v_ (8)

where α^m^_v_ is the alpha modifier for a viral load strata *v*

*Infected partner receiving treatment (no alpha modifying interventions):*

VL_treat_ = VL - (VLdec * P_adh_); V_treat_ is then assigned a new VL strata *v’*

α^mt^_v_  is the alpha modifier of the new VL strata on treatment *v’*

The final alpha value is then calculated as in equation 8 above

For conditions where specific interventions are implemented see section on Interventions below.

To calculate β we assumed a binomial process where the number of trials referred to the number of shared injections per partnership and the probability of successful transmission is described by α^i^_v_ as above. Therefore, beta was calculated using the following equation.

β^i^_V_ = 1 -(1 - α^i^_V_)^SHARED_INJECTIONS_PER_YEAR/IDU_PARTNER_CHANGE_RATE^

Finally for any given interaction between an infected compartment and a susceptible compartment with risk characteristics *rR* the total force of infection was calculated as:

Total force of infection = λ^t^= λ^s^ + λ^I^ (9)

**Table S2.** Input parameters

| **Sexual risk characteristics** |  | **Value** | **Range** | **Source** | |
| --- | --- | --- | --- | --- | --- |
| **P_abs_** | Proportion of population who are abstinent | 21.0% | 17.0-32.0% | Adiamora, et al 2007 | |
| **Pr_i=1,l=2,k=2_** | Probability of monogamous relationship (if sexually active)—Heterosexual Men | 78.2% | … | NYC Community Health Survey [[4](#_ENREF_4)] | |
| **Pr_i=1,l=3,k=2_** | Probability of multiple partnerships (if sexually active)—Heterosexual Men | 21.8% | 16.1- 23.6% | [[4](#_ENREF_4)] | |
| **Pr_i=2,l=2,k=2_** | Probability of monogamous relationship (if sexually active)—Homosexual Men | 55.8% | … | [[4](#_ENREF_4)] | |
| **Pr_i=2, l=3,k=2_** | Probability of multiple partnerships (if sexually active)—Homosexual Men | 44.2% | 25.6-63.6% | [[4](#_ENREF_4)] | |
| **Pr_i=1,l=2,k=1_** | Probability of monogamous relationship (if sexually active)—Heterosexual Women | 91.1% | … | [[4](#_ENREF_4)] | |
| **Pr_i=1,l=3,k=1_** | Probability of multiple partnerships (if sexually active)—Heterosexual Women | 8.9% | 6.9-10.4% | [[4](#_ENREF_4)] | |
| **Pr_i=2,l=2,k=1_** | Probability of monogamous relationship (if sexually active)—Homosexual Women | 48.9% | … | [[4](#_ENREF_4)] | |
| **Pr_i=2,l=3,k=1_** | Probability of multiple partnerships (if sexually active)—Homosexual Women | 51.1% | … | [[4](#_ENREF_4)] | |
| **Po_k=2,i=2_** | Proportion of Men who are MSM exclusively | 4.8% | 2-10% | [[4](#_ENREF_4)] | |
| **Po_k=2,i=3_** | Proportion of Men who are bisexual | 0.8% | … | [[4](#_ENREF_4)] | |
| **Po_k=2,i=1_** | Proportion of Men who are heterosexual | 94.4% | … | [[4](#_ENREF_4)] | |
| **Po_k=1,i=2_** | Proportion of Women who are homosexual | 1.3% | … | [[4](#_ENREF_4)] | |
| **Po_k=1,i=3_** | Proportion of Women who are bisexual | 1.1% | … | [[4](#_ENREF_4)] | |
| **Po_k=1,i=3_** | Proportion of Women who are heterosexual | 97.6% | … | [[4](#_ENREF_4)] | |
| **Intravenous Drug Use Characteristics** |  |  |  |  | |
| **Pi** | Proportion of population that uses IV drugs | 1.43% (143 per 10,000 pop) | 1.33-1.91% | Brady 2008[[5](#_ENREF_5)] | |
| **Pi_risk_** | Proportion of IDU that have unsafe injection practices | 32% | 15%-50% | NHBS NYC Data 2009[[6](#_ENREF_6)] | |
| **Pi_k=2_** | Proportion of IDUs that are Men | 70% | … | NHBS NYC Data 2009[[7](#_ENREF_7)] | |
| **Sexual and IDU transmission** |  |  |  |  | |
| **α^s^_k=2,K=2_** | Transmission risk per sex act (MSM) | 0.00167 | … | Baggaley R 2010 [[8](#_ENREF_8)] | |
| **α^s^_k=2,K=1_** | Transmission risk per sex act (F🡪M) | 0.00042 | … | Boily M 2008 [[9](#_ENREF_9)] | |
| **α^s^_k=1,K=2_** | Transmission risk per sex act (M🡪F) | 0.00081 | … | [[9](#_ENREF_9)] | |
| **α^i^** | Transmission risk per unsafe needle sharing act | 0.003 | … | Tokars JL 1993 [[10](#_ENREF_10)] | |
| **α^m^_v=0_** | Relative risk of transmission if VL category 0-2.5 log copies/ml | 0.16 | … | Attia S 2009 [[11](#_ENREF_11)] | |
| **α^m^_v=1_** | Relative risk of transmission if VL category 2.5-3.5 log copies/ml | 1.87 | … | [[11](#_ENREF_11)] | |
| **α^m^_v=2_** | Relative risk of transmission if VL category 3.5-4.5 log copies/ml | 6.54 | … | [[11](#_ENREF_11)] | |
| **α^m^_v=3_** | Relative risk of transmission if VL category 4.5-5.5 log copies/ml | 8.85 | … | [[11](#_ENREF_11)] | |
| **α^m^_v=4_** | Relative risk of transmission if VL category >5.5 log copies/ml | 9.03 | … | [[11](#_ENREF_11)] | |
| **APP** | Sex acts per partnership | 89 | 50-100 | Mosher WD 2005 [[12](#_ENREF_12)] | |
|  | Shared injections per year | 70 | 25-100 | Expert opinion | |
| **c_IDU_** | IDU needle partner change rate | 5 | 2-10 | Expert opinion | |
| **C*_l=1_*** | Monogamous partnership change rate (*l=1*) | 1 | N/A |  | |
| **C*_l=2_*** | Multiple partnership (*l*=2) change rate | 3 | 2-5 | Expert opinion | |
| **ε** | Degree of assortative sexual mixing | 0.7 |  | Expert opinion | |
| **HIV risk behaviors and biological/behavioral modifiers of transmission** |  |  |  |  | |
| **P_STI_** | Prevalence of untreated STI | 6.9% | 0.1-10% | Epiquery [[13](#_ENREF_13)]; Benedetti J 1994 [[14](#_ENREF_14)] | |
| **P_circ_** | Prevalence of no circumcision | 43.9% | 38.8-49% | NYC HANES 2008[[15](#_ENREF_15)], McKinney 2008[[16](#_ENREF_16)] | |
| **P_alc_** | Prevalence of unhealthy alcohol use | 5% | 2-10% | Wunsch-HItzig[[17](#_ENREF_17)] | |
| **P_condom_** | Prevalence of consistent condom usage | 35% | 20-50% | [[4](#_ENREF_4)] | |
| **P_cofactor_** | Prevalence of any risk cofactor^1^ |  | … |  | |
|  | Relative risk of alcohol use on condom nonuse | 1.45 | 1.31-1.60 | Shuper 2009 [[18](#_ENREF_18)] | |
|  | Relative risk of alcohol use on ART nonadherence | 2.13 | 1.77-2.49 | Braithwaite 2005 [[19](#_ENREF_19)] | |
|  | Relative risk of alcohol use on multiple sex partners | 1.64 | 1.11-2.36 | Adimora 2011[[20](#_ENREF_20)] | |
| **A^p^_condom_** | Relative risk reduction of HIV seroconversion when using condoms | 80% | 35-94% | Cochrane Database Syst Rev 2002[[21](#_ENREF_21)] | |
| **A^p^_proph_** | Relative risk reduction of HIV seroconversion when using PEP | 80% | 15-63% | Cardo DM [[22](#_ENREF_22)]1997 | |
| **A^p^_STI_** | Relative risk reduction of HIV seroconversion if treated STI | 40% | 30-80% | Grosskurth H 1995 [[23](#_ENREF_23)] | |
| **HIV disease related** |  |  |  |  | |
| **P_test_** | Probability of annual HIV test | 31% | 20%-50% | [[4](#_ENREF_4)] | |
| **P_LTC_** | Probability of linkage to care | 75% | … |  | |
| **P_ART_** | Probability of initiating ART if in care | 88% | 65-95% | Expert opinion | |
| **CD4/CD4SD** | Mean CD4 /SD for initialization | 350/200 | … | Need reference | |
| **VL/VLSD** | Mean VL /SD for initialization (untreated HIV) | 4.45/0.78 | … | Need reference | |
| **VL’/VLSD’** | Mean/SD VL for initialization (treated HIV) | 2.45/0.78 | … | Need reference |  |
| **η** | Rate of transition between acute HIV infection and steady state/chronic HIV infection | 8.7^Δ^ | … | Expert opinion | |
| **VLdec** | Average log VL decrement if on ART | 2.41 | … | Braithwaite RS 2007 [[24](#_ENREF_24)] | |
| **Padh** | Probability of adherence (% doses taken) | 63% | 50%-85% | Braithwaite RS 2007 [[24](#_ENREF_24)] | |
| **Madh_y_** | Relative risk of death given adherence % of y (0-100%) [Base case mean adherence assumed to be 63%] | 4.622 (0); 1.607 (10%); 1.305 (20%)  1.165 (30%); 1.080 (40%); 1.033(50%);  1.002(60%); 0.993 (70%); 0.984 (80%); 0.979 (90%); 0.974 (100%) |  | Derived from Braithwaite RS HIV progression model | |
| **Demographics** |  |  |  |  | |
| **μ_r_** | Age-related mortality rate | 0.0068 (6.8/1000 pop) |  | NYC vital statistics [[25](#_ENREF_25)] | |
| **θ** | Fertility rate | 0.0156 (15.6/1000 pop/year) |  | [[25](#_ENREF_25)] | |
| **D_a=0_** | Duration of childhood | 13 years |  | Assumption | |
| **D_a=1_** | Duration of adulthood | 62 years |  | Assumption | |

**^Δ^ Rate= 1/duration = 1/ avg time spent in acute infection 6 weeks(0.115 years) = 1/.115 = 8.7**

1. **HIV Progression Model**

HIV disease progression was modeled by the inclusion of stationary rates developed from a previously developed and validated computer simulation. [[1](#_ENREF_1)] {Braithwaite, 2005 #190}Within the epidemic compartmental model CD4 count was categorized into five mutually exclusive compartments **(*cd*)** [0-50, 50-200, 200-350, 350-500, >500 cells/mm^3^. Viral load was categorized similarly **(*v*)** [0-2.5, 2.5-3.5, 3.5-4.5, 4.5-5.5, >5.5 log copies/ml]. Upon initialization, compartments representing PLWHA in care and treatment were assigned a CD4 count and VL as represented in distribution (Q’) described in Figure S3a-b. Those compartments representing PLWHA not on treatment were determined by distribution (Q) described in Figure S3c-d.

These distributions describing the CD4 and VLs for PLWHA at the start of the simulation were created by calculating the cumulative distribution function [[26](#_ENREF_26)] for CD4 (using inputs of CD4 mean and SD and the CD4 category values of strata), VL (using inputs of VL mean, SD and category values of strata) and multiplying these probabilities.

All persons infected with HIV after the start of the simulation were assigned a CD4 strata of >500 cells/mm^3^ and a viral load from a normal distribution with a mean of 4.45 log copies and standard deviation of 0.78.

**Figure S3a-d**. Probability distributions for initialization of CD4 count strata and VL strata within HIV infected compartments

1. Probability distribution (Q’*_cd,v_*) of CD4 and VL categories for HIV infected persons on ART at initialization

|  |  | CD4 Count Category/Compartment (*c*) | | | | |
| --- | --- | --- | --- | --- | --- | --- |
|  | Category (probability) | **0-50** | **50-200** | **200-350** | **350-500** | **>500** |
| Viral Load Category/ Compartment (*v*) | **0-2.5** | 3.51% | 8.40% | 14.37% | 14.37% | 11.91% |
|  | **2.5-3.5** | 2.57% | 6.16% | 10.53% | 10.53% | 8.73% |
|  | **3.5-4.5** | 0.57% | 1.36% | 2.32% | 2.32% | 1.92% |
|  | **4.5-5.5** | 0.03% | 0.07% | 0.12% | 0.12% | 0.10% |
|  | **>5.5** | 0.00% | 0.00% | 0.00% | 0.00% | 0.00% |

1. Density map of probability distribution Q’


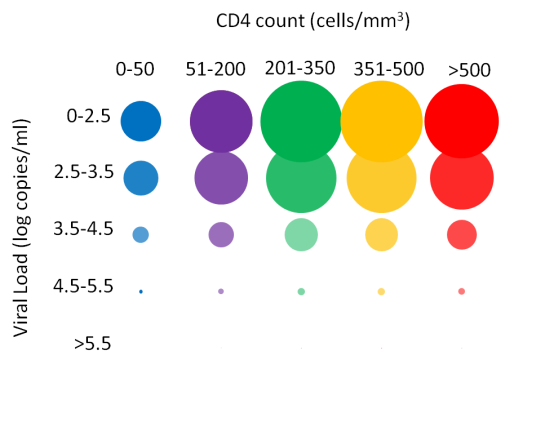


1. Probability distribution (Q*_cd,v_*) of CD4 and VL categories for HIV infected persons not on treatment at initialization

|  |  | CD4 Count Category/Compartment (*c*) | | | | |
| --- | --- | --- | --- | --- | --- | --- |
|  |  | **0-50** | **51-200** | **201-350** | **351-500** | **>500** |
| Viral Load Category or Compartment (*v*) | **0-2.5** | 0.04% | 0.70% | 2.77% | 2.57% | 0.60% |
|  | **2.5-3.5** | 0.10% | 1.68% | 6.62% | 6.16% | 1.42% |
|  | **3.5-4.5** | 0.17% | 2.88% | 11.32% | 10.53% | 2.44% |
|  | **4.5-5.5** | 0.17% | 2.88% | 11.32% | 10.53% | 2.44% |
|  | **>5.5** | 0.14% | 2.39% | 9.38% | 8.73% | 2.02% |

1. Density map of probability distribution Q*_cd,v_*


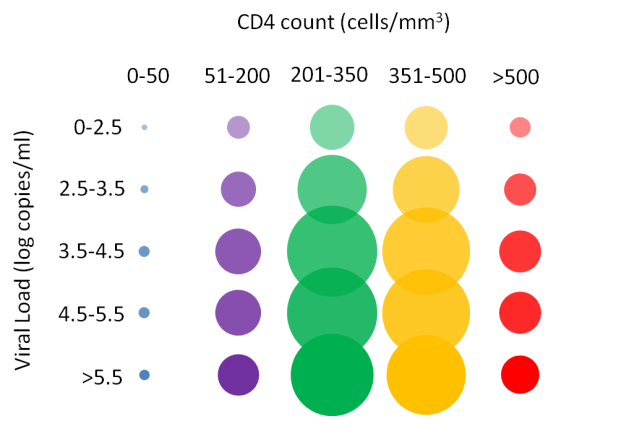


- 1. Integration with epidemic model

The rate of transition between these compartments ([*cd,v*]_(t)_ 🡪 [*cd,v*] _(t+1))_; **π**_cd1v1cd2v2,_ hereafter shortened to **π_cd,v_**) were determined by referencing rate transition tables under two different conditions (on ART and off ART) depending on ART threshold (assumed to be <500 cells/mm^3^ under base case). These rate transition tables were generated from the previously mentioned disease progression model.

This was achieved by using the previously mentioned Braithwaite stochastic HIV progression model to determine the rates of transition between CD4 count and viral load categories, and then substitute these inferred rates into a deterministic model. More specifically, two separate one million trial simulations were conducted (under conditions of no ART available and ART available), to generate “off-care” and “on-care” estimates of disease progression. During these simulations state transitions between the five aforementioned viral load categories and CD4 categories were tracked, as well as transitions between any combination of CD4 and VL and HIV-related death. Rates were then calculated and “lookup tables” generated that indexed these state transitions by current CD4, VL, ART status, and HIV-related death. (yes/no).

We evaluated the validity of this construction by subjecting the compartmental model to a similar analysis as that performed in a previous published analysis [[27](#_ENREF_27)] using the Braithwaite stochastic model after harmonizing the assumptions and inputs between the two models. Results of the comparison between different monitoring strategies were similar between equivalent simulations (stochastic model vs transmission model). In addition, the rank order of strategies from most effective to least was nearly identical between the two different models. We concluded, therefore, the deterministic model mimics the mean behavior of the stochastic model.

Progression through the spectrum of engagement was modeled as a stepwise dependent process starting from HIV uninfected through HIV infected and on ART (Figure S1). Initiation of ART was simulated when CD4 category fell beneath stated ART threshold and corresponded to the utilization of the “on care” integrated look-up table. Mortality from HIV (if infected) (μ*_cd,v_*) could occur from any infected compartment while mortality unrelated to HIV (μ_age_) could occur from any compartment. Under conditions of treatment, transitions between CD4, VL strata are referred to as π’_cd,v_  and AIDS related mortality rates as μ’_cd,v_. HIV-related mortality rates (μ_cd,v_ or μ’_cd,v_) were determined from indexing the appropriate HIV progression model lookup tables. Drug resistance was not accounted for within this model.

1. **HIV Prevention pathways and interventions**

HIV prevention interventions were hypothesized to affect one or more “pathways” modifying HIV sexual or needle based transmission (Figure S4). The specific “mapping” of interventions and their pathways was developed through a collaborative discussion between the academic modeling group and the Department of Health and Mental Hygiene.

An intervention can affect specific pathways that influence HIV transmission in one of four direct mechanisms in the model, a) applying a modifier to alpha (probability of transmission during sex or injection act between discordant pair); b) modifying/shifting proportions of relevant compartments; c) modifying implicit rates/probabilities d) direct modifier on HIV – related mortality rates. The duration of any activated intervention and its effect in any specified model run was considered as binary (on/off) and remains so throughout the model time horizon.

Every intervention had a point estimate (a relative risk or risk reduction) for its effect size (τ) on each pathway, an upper and lower bound to represent the uncertainty of these estimates, and a target group (Table S3). The magnitude of the effect size affecting one pathway is the same regardless of the specific intervention (except where explicitly noted) that activates it. If an intervention was thought not to affect a given pathway a null effect size was assumed and no bounds applied. One or more interventions could be “activated” during a simulation. We assumed that if more than one intervention “activated” a specific pathway in a given target group the effect size would only apply once.

Effect sizes of interventions on “mapped” pathways were taken or derived from relevant literature sources. In cases where no relative risk (RR) was reported (e.g. odds ratios were used) review of the primary source and calculation of a relative risk (if applicable) was undertaken. If sufficient data was unavailable in primary source to calculate a RR then a conversion from odds ratio to risk ratio/relative risk was estimated using a published method.[[28](#_ENREF_28)]. Where appropriate probabilities were converted to rates (r = - [ln (1 – P)] / t) and vice-versa (p = 1 – exp(-rt)).

**Figure S4a-b.** HIV transmission “pathways” that are influenced by prevention interventions

- 1. Schematic of constructs in transmission simulation and pathways which impact HIV transmission.

b. Pathway mechanisms

| **Pathway** | **Lower risk -barrier :**  **Condom use** | **Lower risk -nonbarrier:**  **Prevalence of non-HIV STI’s** | **Lower risk non-barrier-**  **Prophylactic ART (PEP)** | **Needle sharing** | **Number of sexual partners** | **IV drug use** | **Earlier ART: HIV testing** | **Earlier ART: HIV linkage** | **Effective ART: Adherence to ART** |
| --- | --- | --- | --- | --- | --- | --- | --- | --- | --- |
| **Intervention “mapped” to this pathway** | Impacts probability of consistent condom use | Impacts proportion of population with untreated STI | Impacts proportion of population using prophylaxis | Impacts proportion of IDUs that practice unsafe injection techniques | Impacts proportion of persons in multiple partnering compartment | Impacts proportion of population that are IDUs | Impacts probability of annual testing for HIV | Impacts probability of transition from HIV infected to HIV in care | Impacts probability of nonadherence among those on ART |
| **Model mechanism** | Sexual alpha modifier | Sexual alpha modifier | Sexual alpha modifier | Injection drug use alpha modifier | Shift compartment proportions | Shift compartment proportions | Rate/Transition  modifier | Rate/Transition  Modifier | Progression modifier |

**Table S3.** ECHPP interventions, HIV transmission pathway mapping and targeted populations

***a****. Legend for part b -“pathways and target population”. Percentages represent proportions of total population* ***b.*** *Mapping of interventions to HIV transmission pathways and the prioritization of these interventions. An intervention will activate pathway(s) (grey arrows; primary effect highlighted in box) within a specific target population.* ***High-risk*** *refers to proportions of the population who exhibit one or more of the following characteristics: multiple sexual partners, IDU, or one of the following cofactors (alcohol abuse, non-IV drug abuse mental health).*

HIV negative **(98.2%)**

HIV positive (1.8%)

High risk

(dotted circle)

HIV known

(grey circle)

90% (of HIV+)

12%

0.375%

**a.)**

**b.)**

| CDC Intervention | Descriptor | Pathways^1^ and Target population | Primary effect size and bounds for sensitivity analysis | Cost-per-unit and bounds for sensitivity analysis |
| --- | --- | --- | --- | --- |
| Routine opt-out screening for clinical settings | TESTING-CLINICAL | 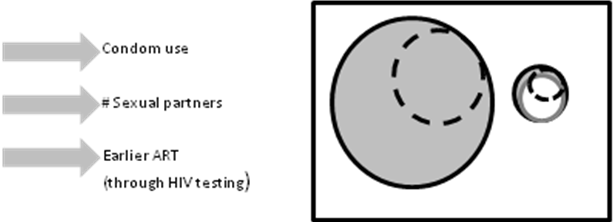 | 33% (30-40%) increase in probability of annual HIV testing[[29](#_ENREF_29),[30](#_ENREF_30),[31](#_ENREF_31)] | $100 ($37.5-147) |
| HIV testing in non-clinical settings to identify undiagnosed HIV infection | TESTING-NON-CLINICAL | 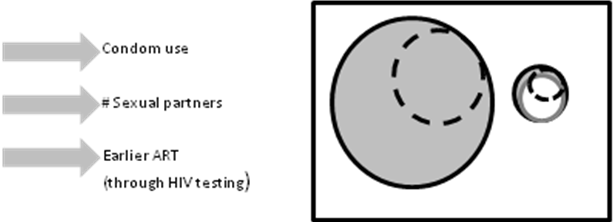 | 10% (2-19%) increase in probability of annual HIV testing[[32](#_ENREF_32),[33](#_ENREF_33)] | $121 ($109-162) |
| Condom distribution prioritized to target HIV-positive persons at highest risk of transmitting HIV infection | CONDOMS-HR, HIV+ | 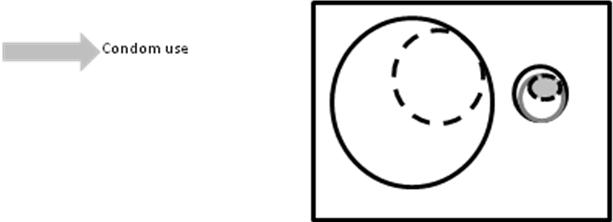 | 12% (3-21.5%) increase in probability of consistent condom use[[34](#_ENREF_34)] | $0.07 per condom ($0.05-1.00) |
| Condom distribution prioritized to target HIV-negative persons at highest risk of acquiring HIV infection | CONDOMS –HR, HIV- | 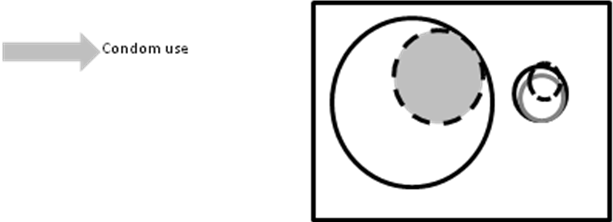 | 12% (3-21.5%) increase in probability of consistent condom use[[34](#_ENREF_34)] | $0.07 per condom ($0.05-1.00) |
| Condom distribution for the general population | CONDOMS-GENERAL | 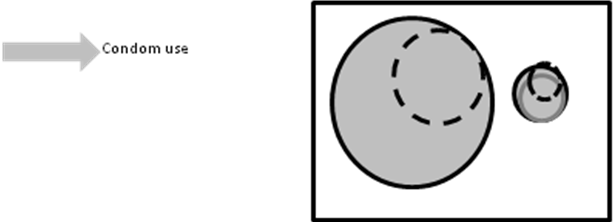 | 12% (3-21.5%) increase in probability of consistent condom use[[34](#_ENREF_34)] | $0.07 per condom ($0.05-1.00) |
| Provision of Post-Exposure Prophylaxis to populations at highest risk | PEP – HR, HIV- | 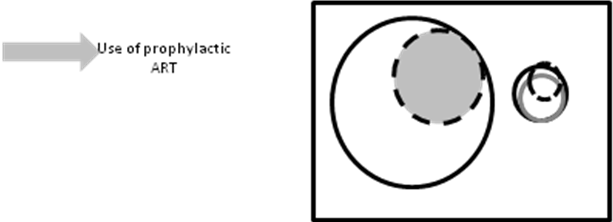 | 42% (25-70%) increase in probability of any use of prophlyactic ART[[35](#_ENREF_35)] | $2625 ($1312-3938)) |
| Provision of Post-Exposure Prophylaxis to population at risk | PEP | 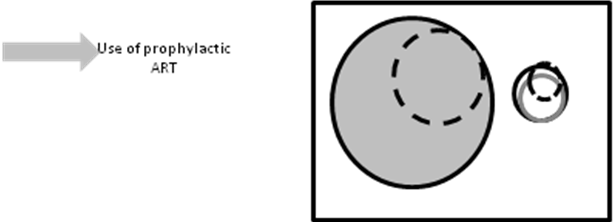 | 42% (25-70%) increase in probability of any use of prophlyactic ART[[35](#_ENREF_35)] | $2625 ($1312-3938) |
| Implement linkage to HIV care, treatment, and prevention services for those testing HIV positive and not currently in care | LINKAGE TO CARE | 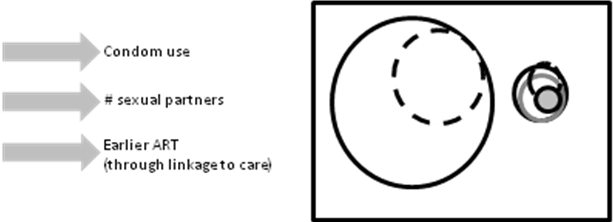 | 30% (9-37.5%) increase in probability of linkage to care once diagnosed[[36](#_ENREF_36)] | $1251 ($1078- 1424)[[37](#_ENREF_37)] |
| Implement interventions or strategies promoting adherence to antiretroviral medication and retention in care for HIV-positive persons | CARE COORDINATION | 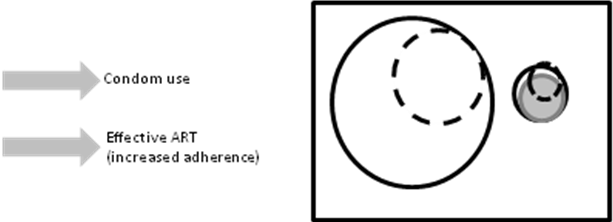 | 20% (7.5-32%) increase in adherence[[38](#_ENREF_38),[39](#_ENREF_39)] | $6000($3000-9000) |
| Implement STD screening according to current guidelines for HIV-positive persons | STD SCREENING HIV+ | 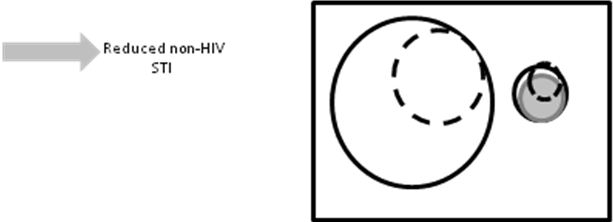 | 30% (20-80%) reduction in non-HIV STI[[23](#_ENREF_23)] | $230 ($178-230) |
| Implement STD screening according to current guidelines for HIV-positive persons | STD SCREENING HR, HIV+ | 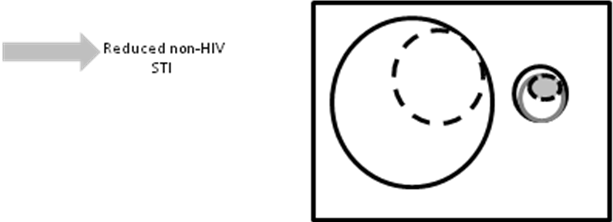 | 30% (20-80%) reduction in non-HIV STI[[23](#_ENREF_23)] | $230 ($178-230) |
| Implement STD screening according to current guidelines for all persons | STD SCREENING ALL | 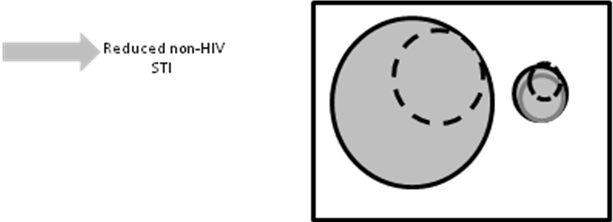 | 30% (20-80%) reduction in non-HIV STI[[23](#_ENREF_23)] | $230 ($178-230) |
| Implement ongoing partner services for HIV-positive persons | PARTNER SERVICES | 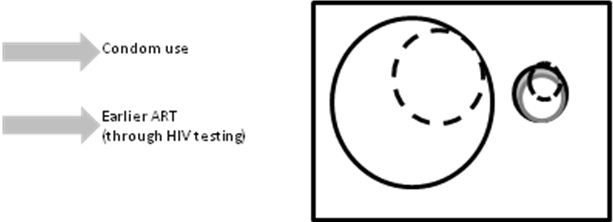  Proportion of HIV undetected persons become detected | 2.8% (2-5%) increase in undetected HIV testing and entering care[[40](#_ENREF_40)] | $1496 ($748-2244) |
| Behavioral risk screening followed by risk reduction interventions for HIV-positive persons (including those for HIV-discordant couples) at risk of transmitting HIV | RISK REDUCTION-HIV+ | 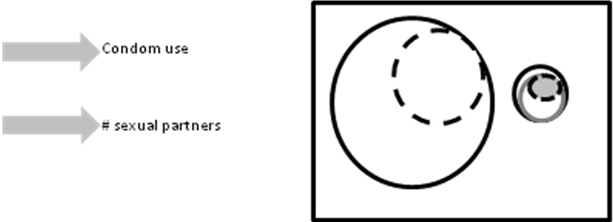 | 25% (1-50%) decrease in persons engaging in multiple, concurrent partnerships[[41](#_ENREF_41)] | $1906 ($1000-2803) |
| Implement linkage to other medical and social services for HIV-positive persons | LINKAGE TO SUPPORT-HIV+ | 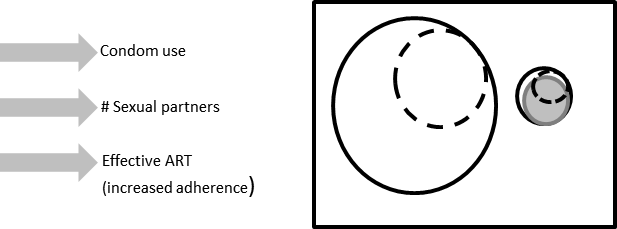 | see above for each component | $796 ($398-1194) |
| HIV and sexual health communication or social marketing campaigns targeted to relevant audiences | SOCIAL MARKETING-HIV+ | 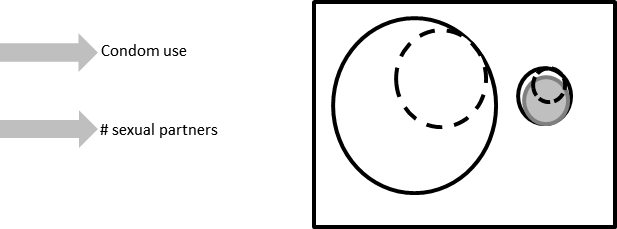 | see above for each component | $8.60 ($4-13) |
|  | SOCIAL MARKETING-HR, HIV- | 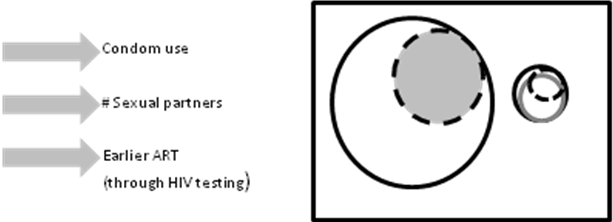 | see above for each component | $8.60 ($4-13) |
|  | SOCIAL MARKETING-General | 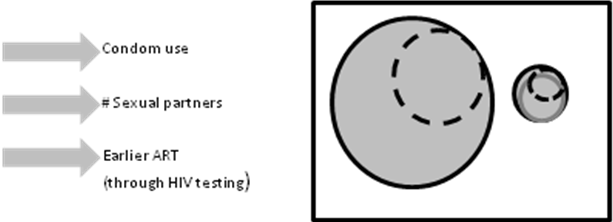 | see above for each component | $8.60 ($4-13) |
|  | SOCIAL MARKETING-Providers | 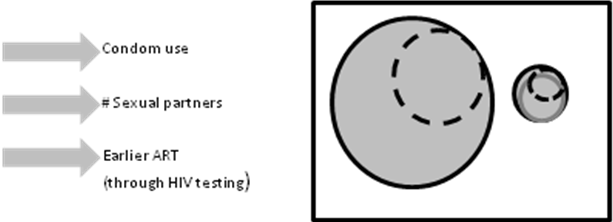 | see above for each component | $8.60 ($4-13) |
| Evidence based community interventions that reduce HIV risk | COMMUNITY LEVEL INTERVENTION | 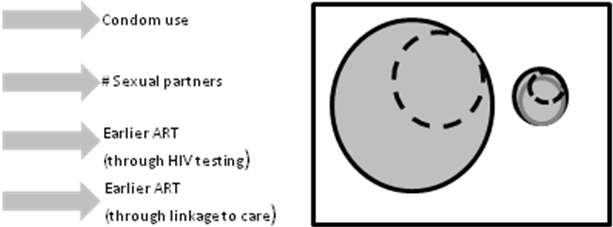 | see above for each component | $0.75 ($0.37-1.10) |
| Targeted use of HIV and STD surveillance data to prioritize risk reduction counseling and partner services for persons with previously diagnosed HIV infection with a new STD | TARGETED SURVEILLANCE | 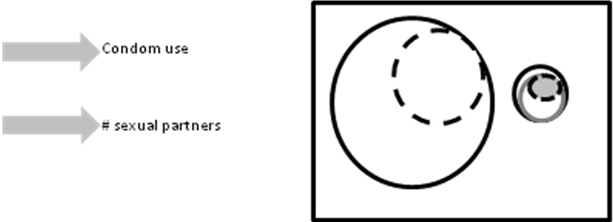 | see above for each component | $104.54 ($52-157) |
| For HIV-negative persons at highest risk , linkages to social factors impacting HIV incidence | SOCIAL SERVICES-HR, HIV- | 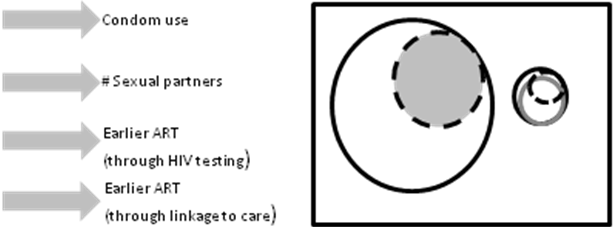 | see above for each component | $175.39 ($88-263) |
| Brief alcohol screening and interventions (SBIRT) | SBIRT-HIV+^2^ | 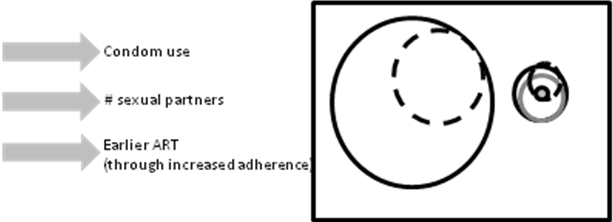  Decreases prevalence of unhealthy alcohol users in HIV+ population | 10% (5-20%) decrease in unhealthy alcohol use | $100 ($54.62- 155.66) |
|  | SBIRT-HR, HIV(-)^2^ | 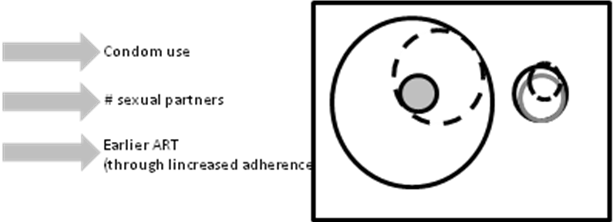  Decreases prevalence of unhealthy alcohol users in high risk HIV negative population | 10% (5-20%) decrease in unhealthy alcohol use[[42](#_ENREF_42)] | $100 ($54.62- 155.66) |
| Brief screening and interventions aimed at different risk characteristics | COFACTORS- HIV+, +cofactors^3^ | 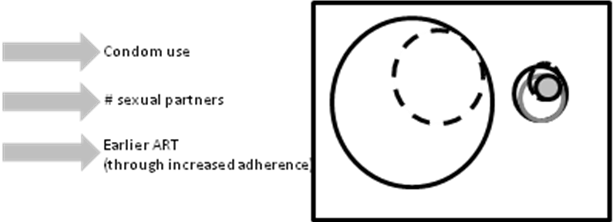  Decreases prevalence of other cofactors in HIV positive population | 10% (5-20%) decrease in any cofactor prevalence | $100 ($54.62- 155.66) |
|  | COFACTORS-HR, HIV-, + cofactors^3^ | 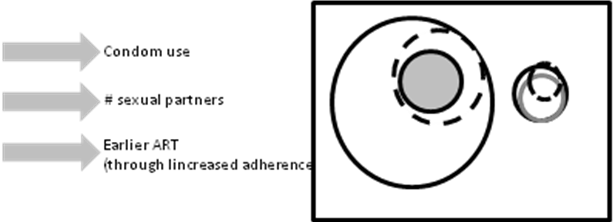  Decreases prevalence of other cofactors in high risk, HIV negative population | 10% (5-20%) decrease in any cofactor prevalence | $100 ($54.62- 155.66) |

^1^ Where no specified pathways present (e.g. partner services intervention) a simple description of intervention’s effect is given.

^2^Includes proportion of high risk persons who are alcohol abusers

^3^Includes proportion of high risk persons who are not alcohol abusers

3.4.1 Interventions which modify transmission probability per act (α)

Recall from above the calculation for transmission probability is dependent upon partnership type and viral load of infected partner. Several of the considered interventions act along pathways that directly modify the transmission probability per act (green highlighted pathways in Figure S4b). To calculate the adjusted alpha under the intervention scenario, an alpha modifier related to the intervention is first calculated (α^m^_INT_).

α^m^_INT_= ((1-P_b_) – ((1-P_b_)*(1-τ)))*A^p^ + (1-((1-P_b_)-((1-P_b_)*(1-τ))))*1 (10)

Where P_b_= baseline probability; τ = effect size of intervention; A^p^= alpha multiplier of pathway

For example:

Assume condom distribution intervention activated which has an effect size of 12% (i.e. probability of using condoms consistently in target population is 12% greater), baseline probability of consistent condom use is 35% and the alpha modifier of condom use during sex is 0.2 (an 80% risk reduction in transmission).

α^m^_INT_= ((1-0.35) – ((1-0.35)*(1-0.12)))*0.2 + (1-((1-0.35)-((1-0.35)*(1-0.12))))*1 = 0.938

The final transmission probability then equals:

α_kKV_ = α_kK_ * α^m^_v_ * α^m^_INT_ (11)

- - 1. Interventions that modify compartment populations

The shifting of proportions of specific compartments resulting from activation of interventions that act along these pathways is calculated as follows:

N_r_’=N_r_ * τ_INT_ (12)

where N_r_ refers to the total number within a specific compartment with risk characteristics r; τ is intervention effect size.

N_r_’ persons will move from specified risk strata usually to different risk strata depending on the specific intervention. For those interventions influencing the sexual partnering pathway , N_r_ refers to compartments where *l=3* (multiple partners). The resultant N_r_’ from the above equation are moved to relevant *l=2* compartments. Similarly for any interventions mapped to the IDU reduction pathway the N_r_ refers to all IDU compartments (j=2) and the calculated N_r_’ are moved to relevant non-IDU( j=1) compartments.

3.4.3 Interventions that modify transition rates along spectrum of engagement in care

The effect of interventions that act via pathways which modify ν are calculated by applying the intervention effect sizes (τ_test_ or τ_link_) to the assumed probabilities of either HIV testing (P_test_) and or linkage to care (P_LTC_)as follows:

ν’ = (1-(1-P_test_*1-τ_test_ ))*(1-(1-P_LTC)_*(1-τ_link_)) (13)

For example:

Assume a testing intervention is implemented that has an effect size of 33% (i.e. increases annual probability of HIV testing by 33%; linkage to care probability unchanged). The baseline probability of HIV testing is 31% and the baseline probability of linkage to care is 74%.( ν= 0.23; 23%)

ν’ = (1- (1-0.31)*(1-0.33)) * (1- (1-0.74) * 1)) = ~ 0.40; 40%

- - 1. Interventions that modify adherence to ART

Interventions that modify adherence to ART act by modifying the HIV-related mortality rate at a specified CD4 and VL combination. The probability of adherence under conditions when such an intervention is active is calculated by:

P_adh_’ = 1 - ((1-P_adh_) * (1-τ_int_)) (14)

A modifier that defines the relative risk of mortality at a given level of adherence ([Madh_y_] see Table S2) is then applied to the HIV-associated mortality rate (μ’_cd,v_). If the value of P_adh_’ is between two values a linear interpolation is performed.

μ_INT_= μ_cd,v_ * Madh_y_ (15)

For example:

Assume an adherence intervention is implemented that has an effect size of 20% (i.e. probability of adherence is increased by 20%, or conversely non-adherence is decreased by 20%). The baseline probability of adherence is 63%.

P_adh_’ = 1- ((1-0.63)*(1-0.20)) = 0.70 ~ 70%

From Table S2 Madh_y_ =0.993 then μ_INT_ = μ_cd,v_ * 0.993

1. **Initialization of population matrix**

We calculated the initial populations of each compartment in the model using New York City specific epidemiological data for both the HIV negative and HIV positive populations[[4](#_ENREF_4),[7](#_ENREF_7),[13](#_ENREF_13),[15](#_ENREF_15)]. We synthesized data regarding the epidemiology of HIV infection from New York City based surveillance[[43](#_ENREF_43)] [Table S4].

**Table S4.** New York City derived HIV data and transmission risks in 2009

| Subgroup | Male HIV positive  (known)^#^ | Male HIV negative | Female HIV positive (known)^#^ | Female HIV negative | Total HIV positive  (known) | Total HIV negative |
| --- | --- | --- | --- | --- | --- | --- |
| Adults (13-65) | 76,770 | 2,619,632 | 31,596 | 2,806,670 | 108,366 | 5,426,302 |
| *Transmission Risk factor*** | | | | |  | |
| Heterosexual | 5,637 (7%) | N/A | 15,081 (48%) | N/A | 20,718 (19%) | N/A |
| Homosexual | 35,882 (47%) | N/A |  | N/A | 35,882 (33%) | N/A |
| IDU | 15,051 (20%) | N/A | 6,151 (19%) | N/A | 21,202 (20%) | N/A |
| Children (0-12) | 243* | 716,464 | 277* | 685,579 | 520 | 1,402,043 |
| Totals | 77,013 | 3,336,096 | 31,873 | 3,492,249 | 108,886 | 6,828,345 |
|  | 3,413,109 | | 3,524,122 | | 6,937,231 | |

^#^ HIV+ population at time=0 is equal to known population (input) + estimated fraction of unknown community HIV+ infected persons

* Calculated/derived value

** Proportion of adults with HIV with determined /reported transmission risk factor. Proportions do not equal 100% because of populations of persons with unknown transmission risk. The unknown population is distributed across risk factor compartments in proportion.

The initialization of the HIV infected population was performed by distributing the reported PLWHA in NYC across the spectrum of care with reference to proportions of PLWHA in care and or on treatment derived from NYC estimates. To scale up the initial population of HIV infected to represent the hypothesized true prevalence of HIV in NYC (HIV known/reported + HIV undetected) we increased the total number of HIV infected persons by 11% as this was a representative value of the proportion of PLWHA who remain undetected, as it was an average value between two different estimates [[44](#_ENREF_44),[45](#_ENREF_45)] and it provided the best fit of HIV prevalence, incidence (new diagnoses) during the validation of the model.

The initial population of the model is outlined in Table S5. Distributions across sexual activity classes (*l*) can be calculated by multiplying a given cell with its respective probability conditional on gender and sexual orientation.

**Table S5.** Calculated initial population distribution across risk strata and HIV infection spectrum of care/engagement

| Age  (*a*) | Gender (*k*) | Orientation (*i*) | IDU (*j*) | Susceptible  (HIV -) | Acute HIV | Chronic HIV | HIV in care | HIV on ART |
| --- | --- | --- | --- | --- | --- | --- | --- | --- |
| Child | M | N/A | N/A | 716,463 | 1 | 24 | 42 | 206 |
|  | F | N/A | N/A | 685,578 | 0 | 23 | 49 | 239 |
| Adult | M | Hetero | N | 2,506,720 | 353 | 409 | 1,327 | 6,478 |
|  |  |  | Y | 6,721 | 2 | 1,529 | 1,839 | 8,980 |
|  |  | Homosexual | N | 87,271 | 18 | 5,618 | 6,768 | 33,044 |
|  |  |  | Y | 4,782 | 2 | 1,088 | 1,309 | 6,389 |
|  |  | Bisexual | N | 13,193 | 3 | 965 | 1,381 | 6,741 |
|  |  |  | Y | 946 | 0 | 215 | 259 | 1,264 |
|  | F | Hetero | N | 2,733,231 | 115 | 1,796 | 3,948 | 19,277 |
|  |  |  | Y | 5,609 | 1 | 1,103 | 1,534 | 7,490 |
|  |  | Homosexual | N | 36,705 | 0 | 0 | 0 | 0 |
|  |  |  | Y | 37 | 0 | 7 | 10 | 49 |
|  |  | Bisexual | N | 31,058 | 0 | 0 | 0 | 0 |
|  |  |  | Y | 31 | 0 | 6 | 9 | 42 |
| Totals | | | | 6,828,345 | 495 | 12,808 | 18,523 | 90,437 |
| Total HIV undetected (%) | | | | | 13,303(11%) | |  |  |
| Total HIV infected | | | | | 122,263 | | | |

- 1. Entry and Maturation

Entry into the population was determined by the New York City reported fertility rate for 2008 and was distributed in relative proportion by risk group (r). Entry was assumed at birth but no sexual/IDU activity was assumed to occur until age 13.

Maturation rates were calculated based upon the duration spent in each of the two age categories (D; children, adults). Childhood duration was assumed to encompass from birth to 13 years of age (age of sexual debut under base case). Adult duration encompassed 13-75 years of age. Therefore, maturation rate in any compartment was calculated as 1/duration of age (child or adult). Age-related mortality rate (μ_age_) was considered as a constant and represented the overall published mortality rate per 1000 population for New York City. For any given compartment (not accounting for HIV infection or HIV-related death) entry, maturation and mortality were calculated as follows:

Children:

N_a=0,r_ (*t+1*) = Births*_r_* – ((1/D_a=0_) * N_a=0,r_ (*t*)) − μ_age_  (16)

Adults:

N_a=1,r_ (*t+1*) = ((1/D_a=0_)*N_a=0,r_ *(t)* )) – ((1/D_a=1_) * N_a=1,r_ *(t)* )) - μ_age_ (17)

**5 Outcomes**

The system of nonlinear differential equations were solved numerically to calculate the number of persons in each compartment over time. The following outcome measures were then derived.

- 1. Effectiveness

5.1.1 Total number of HIV infections was calculated at a given time t as:

Σ N _y>1_(t)

Where y refers to status variable (1=susceptible, >1 = HIV infected)

5.1.2 Prevalence at time t was calculated as:

Σ N _y>1_(t) / Σ N(t)

5.1.3 Number of new infections over time horizon T was calculated as:

New infections= ∫^T^ Σ λ_y>1_(t) * N_y=1_(t)dt (18)

5.1.4 Number of infections averted was calculated as:

# of new HIV infections (base case) - # of new HIV infections (intervention scenario)

- 1. Costs

Costs were derived for each considered intervention as a per-person/unit-cost in 2010 US dollars. These costs were generated from NYC DOHMH estimates and in cases where these estimates were not known, literature sources were utilized. These costs represent monies that would need to be budgeted from the perspective of the City of New York and Department of Health and Mental Hygiene in order to roll out or scale up the specified interventions to the entire specified target population. No other costs were considered in the model including payments related to other health care resource utilization such as ART cost, base case testing/counseling, hospitalization, etc. Therefore, there were no calculated costs for the base case scenario (i.e. without incremental interventions implemented). Costs were not discounted.

Total cost for a given intervention scenario at time t was calculated as:

Σ N_int_(t)*cost_int_

where *int* represents the specific interventions activated, N_int_ represents the total population of all targeted compartments for a specific intervention, and cost_int_ the per unit cost of a specific intervention.

Total costs over the time horizon T was calculated as:

Costs= ∫^T^ Σ N_int_(t) dt *cost_int_ (19)

Cost per infection averted was calculated as:

Cost per infection averted = Costs(intervention scenario) / (new infections (base case)- new infection (intervention scenario))

**References**

1. Braithwaite RS, Justice AC, Chang CC, Fusco JS, Raffanti SR, et al. (2005) Estimating the proportion of patients infected with HIV who will die of comorbid diseases. Am J Med 118: 890-898.

2. Garnett GP, Anderson RM (1994) Balancing sexual partnership in an age and activity stratified model of HIV transmission in heterosexual populations. Journal of Mathematics Applied in Medicine & Biology 11: 161-192.

3. Kahn JO, Walker BD (1998) Acute human immunodeficiency virus type 1 infection. The New England journal of medicine 339: 33-39.

4. New York City Department of Health and Mental Hygiene (2009) Community Health Survey.

5. Brady JE, Friedman SR, Cooper HL, Flom PL, Tempalski B, et al. (2008) Estimating the prevalence of injection drug users in the U.S. and in large U.S. metropolitan areas from 1992 to 2002. Journal of urban health : bulletin of the New York Academy of Medicine 85: 323-351.

6. Merchant RC, Seage GR, Mayer KH, Clark MA, DeGruttola VG, et al. (2008) Emergency department patient acceptance of opt-in, universal, rapid HIV screening. Public Health Rep 123 Suppl 3: 27-40.

7. New York City Department of Health and Mental Hygiene (2009) HIV Risk and Prevalence among New York City Injection Drug Users.

8. Baggaley RF, White RG, Boily M-C (2010) HIV transmission risk through anal intercourse: systematic review, meta-analysis and implications for HIV prevention. International Journal of Epidemiology.

9. Boily M-C, Baggaley RF, Wang L, Masse B, White RG, et al. (2009) Heterosexual risk of HIV-1 infection per sexual act: systematic review and meta-analysis of observational studies. The Lancet Infectious Diseases 9: 118-129.

10. Tokars JI, Marcus R, Culver DH, Schable CA, McKibben PS, et al. (1993) Surveillance of HIV infection and zidovudine use among health care workers after occupational exposure to HIV-infected blood. The CDC Cooperative Needlestick Surveillance Group. Ann Intern Med 118: 913-919.

11. Attia S, Egger M, Muller M, Zwahlen M, Low N (2009) Sexual transmission of HIV according to viral load and antiretroviral therapy: systematic review and meta-analysis. AIDS 23: 1397-1404.

12. Mosher WD CA, Jones J (2005) Sexual Behavior and Selected Health Measures: Men and Women 15–44 Years of Age, United States, 2002. Advance Data 362.

13. Blower S, Ma L, Farmer P, Koenig S (2003) Predicting the impact of antiretrovirals in resource-poor settings: preventing HIV infections whilst controlling drug resistance. Curr Drug Targets Infect Disord 3: 345-353.

14. Benedetti J, Corey L, Ashley R (1994) Recurrence rates in genital herpes after symptomatic first-episode infection. Ann Intern Med 121: 847-854.

15. Hygiene NYCDoMHa (2008) New York City health and nutrition examination survey (NYC HANES).

16. McKinney CM, Klingler EJ, Paneth-Pollak R, Schillinger JA, Gwynn RC, et al. (2008) Prevalence of adult male circumcision in the general population and a population at increased risk for HIV/AIDS in New York City. Sexually transmitted diseases 35: 814-817.

17. Wunsch-Hitzig R, Engstrom M, Lee R, King C, McVeigh K (2003) Prevalence and Cost Estimates of Psychiatric and Substance Use Disorders and Mental Retardation and Developmental Disabilities in NYC. New York: New York City Department of Health and Mental Hygiene.

18. Shuper PA, Joharchi N, Irving H, Rehm J (2009) Alcohol as a correlate of unprotected sexual behavior among people living with HIV/AIDS: review and meta-analysis. AIDS and behavior 13: 1021-1036.

19. Braithwaite RS, McGinnis KA, Conigliaro J, Maisto SA, Crystal S, et al. (2005) A Temporal and Dose-Response Association Between Alcohol Consumption and Medication Adherence Among Veterans in Care. Alcoholism: Clinical & Experimental Research 29: 1190-1197.

20. Adimora AA, Schoenbach VJ, Taylor EM, Khan MR, Schwartz RJ (2011) Concurrent partnerships, nonmonogamous partners and substance use among women in the United States. American Journal of Public Health 101: 128-136.

21. Weller SC, Davis-Beaty K (2002) Condom effectiveness in reducing heterosexual HIV transmission. Cochrane Database of Systematic Reviews.

22. Cardo DM, Culver DH, Ciesielski CA (1997) A case-control study of HIV seroconversion in health care workers after percutaneous exposure. NEJM 337: 1485-1490.

23. Grosskurth H, Mosha F, Todd J, al e (1995) Impact of improved treatment of sexually transmitted diseases on HIV infection in rural Tanzania: randomised controlled trial. Lancet 346: 530-536.

24. Braithwaite RS, Kozal MJ, Chang CC (2007) Adherence, virological and immunological outcomes in HIV-infected veterans starting combination antiretroviral therapies. AIDS 21: 1579-1589.

25. New York City Department of Mental Health and Hygiene (2009) Vital statistics.

26. van den Berg T (2011) Digital Spread Option Pricing Model.

27. Braithwaite RS, Nucifora KA, Yiannoutsos CT, Musick B, Kimaiyo S, et al. (2011) Alternative antiretroviral monitoring strategies for HIV-infected patients in east Africa: opportunities to save more lives? J Int AIDS Soc 14: 38.

28. Zhang J, Yu K (1998) What's the relative risk? A method of correcting the odds ratio in cohort studies of common outcomes. JAMA 280: 1690-1691.

29. Anaya HD, Hoang T, Golden JF, Goetz MB, Gifford A, et al. (2008) Improving HIV screening and receipt of results by nurse-initiated streamlined counseling and rapid testing. Journal of General Internal Medicine 23: 800-807.

30. Calderon Y, Cowan E, Nickerson J, et al (2011) Educational effectiveness of an HIV pretest video for adolescents: A randomized controlled trial. Pediatrics 127: 911-916.

31. Mullins TL, Kollar LM, Lehmann C, Kahn JA (2010) Changes in human immunodeficiency virus testing rates among urban adolescents after introduction of routine and rapid testing. Arch Pediatr Adolesc Med 164: 870-874.

32. Rhodes SD, Vissman AT, Stowers J, Miller C, McCoy TP, et al. (2011) A CBPR partnership increases HIV testing among men who have sex with men (MSM): outcome findings from a pilot test of the CyBER/testing internet intervention. Health education & behavior : the official publication of the Society for Public Health Education 38: 311-320.

33. Wilton L, Herbst JH, Coury-Doniger P, Painter TM, English G, et al. (2009) Efficacy of an HIV/STI prevention intervention for black men who have sex with men: findings from the Many Men, Many Voices (3MV) project. AIDS and behavior 13: 532-544.

34. Charania MR, Crepaz N, Guenther-Gray C, Henny K, Liau A, et al. (2010) Efficacy of Structural-Level Condom Distribution Interventions: A Meta-Analysis of U.S. and International Studies, 1998–2007. AIDS and Behavior 15: 1283-1297.

35. Barash EA, Golden M (2010) Awareness and use of HIV pre-exposure prophylaxis among attendees of a seattle gay pride event and sexually transmitted disease clinic. AIDS Patient Care and STDs 24: 689-691.

36. Gardner LI, Metsch LR, Anderson-Mahoney P, et al (2005) Efficacy of a brief case management intervention to link recently diagnosed HIV-infected persons to care. AIDS and Behavior 19: 423-431.

37. Gardner LI, Metsch LR, Anderson-Mahoney P, Loughlin AM, del Rio C, et al. (2005) Antiretroviral Treatment and Access Study Study Group. Efficacy of a brief case management intervention to link recently diagnosed HIV-infected persons to care. AIDS 19: 423-431.

38. Hart JE, Jeon CY, Ivers LC, Behforouz HL, Caldas A, et al. (2010) Effect of directly observed therapy for highly active antiretroviral therapy on virologic, immunologic, and adherence outcomes: a meta-analysis and systematic review. J Acquir Immune Defic Syndr 54: 167-179.

39. Simoni JM, Pearson CR, Pantalone DW, Marks G, N C (2006) Efficacy of interventions in improving highly active antiretroviral therapy adherence and HIV-1 RNA viral load. J Acquir Immune Defic Syndr 43: S23-35.

40. Hogben M, McNally T, McPheeters M, Hutchinson AB (2007) The effectiveness of HIV partner counseling and referral services in increasing identification of HIV-positive individuals a systematic review. American Journal of Preventive Medicine 33: S89-100.

41. Vissers DC, De Vlas SJ, Bakker R, Urassa M, Voeten HA, et al. (2011) The impact of mobility on HIV control: A modelling study. Epidemiol Infect: 1-9.

42. Bertholet N, Daeppen JB, Wietlisbach V, Fleming M, Burnand B (2005) Reduction of alcohol consumption by brief alcohol intervention in primary care: systematic review and meta-analysis. Arch Intern Med 165: 986-995.

43. New York City Department of Health and Mental Hygiene (2009) New York City HIV/AIDS annual surveillance statistics. New York.

44. Begier EM, Bennami Y, Forgione L (2009) Undiagnosed HIV infection among New York City jail entrants, 2006: results of a blinded serosurvey. JAIDS 00: 1-9.

45. Nguyen TQ, Gwynn RC, Kellerman SE (2008) Population prevalence of reported and unreported HIV and related behaviors among the household adult population in New York City, 2004. AIDS 22: 281-287.
