# Supplementary material for: Averting HIV Infections in New York City: A Modeling Approach Estimating the Future Impact of Additional Behavioral and Biomedical HIV Prevention Strategies
Source: PLoS One. 2013 Sep 13;8(9):e73269. doi: 10.1371/journal.pone.0073269 (PMC3772866; doi:10.1371/journal.pone.0073269)
Supplement: File S2 — Supporting text, tables, and figures. Additional results. Figure S5. Efficient frontier of most efficacious packages of HIV prevention strategies in NYC over 20 years. a. Packages (1–7) consist of those combinations of the 16 most effective (as measured by # of infections averted) interventions that have the most favorable incremental cost to effectiveness ratios. All other combinations of the 16 considered interventions fall to the right of the curve and are therefore not preferred. b. Table which provides details on the 7 packages which lie on the efficient frontier including the specific pathways activated by the package of interventions. Figure S6. One way sensitivity analyses for effectiveness and cost-effectiveness ratio of HIV prevention strategies in NYC. a. Range of values for % of HIV averted for each intervention when all input parameters are varied across their spectrum of values that were considered. b. range of values for cost-per-infection averted among those interventions found to be cost-saving under reference case assumptions. Note that STD screening intervention effect size plausible range includes a null effect, therefore, the upper limit of the cost-per-infection averted parameter for this intervention is undefined. Table S6. Alternate time horizons (5, 10 years) of computer simulation and comparative effectiveness of HIV prevention strategies in NYC. Figure S7. Effects of optimization by level of evidence. a. Efficient frontier of combinations of HIV prevention strategies filtered by level of evidence. Packages represent combinations of only those strategies that met or exceeded a specified level of evidence and which had an ICER that was of optimal value. All other combinations fall to the right of the curve and are therefore not preferred (and not shown on the figure). Level A (denoted by a purple triangle) included only those interventions with an evidence grade of A; Level B (denoted by a green square) included interventions with level of e [file pone.0073269.s002.docx]

Supplementary Materials S2:

Additional results

**Additional Results**

**Figure S5a,b.** *Efficient frontier of most efficacious packages of HIV prevention strategies in NYC over 20 years*. **a.** Packages (1-7) consist of those combinations of the 16 most effective (as measured by # of infections averted) interventions that have the most favorable incremental cost to effectiveness ratios. All other combinations of the 16 considered interventions fall to the right of the curve and are therefore not preferred. **b.** Table which provides details on the 7 packages which lie on the efficient frontier including the specific pathways activated by the package of interventions.

a.

b.

Pathways influenced by package of interventions

| Package | Number of HIV infections averted over 20 years | Total cost (millions USD) over 20 years | *More consistent condom usage* | *Lower likelihood of having multiple sexual partners* | *Increased likelihood of HIV testing* | *Increased likelihood of linkage to HIV care* | *Increased likelihood of adherence to HIV treatment* | *Increased likelihood of using post-exposure prophylaxis* | Interventions included in package |
| --- | --- | --- | --- | --- | --- | --- | --- | --- | --- |
| 1 | 5,352 | 19 | X | X |  |  |  |  | Social marketing-HIV+ |
| 2 | 11,562 | 83 | X | X | X | X |  |  | Community level intervention |
| 3 | 19,562 | 1,789 | X | X | X | X | X |  | Community level intervention; Linkage to support-HIV+ |
| 4 | 20,488 | 9,943 | X | X | X | X | X |  | Community level intervention; Linkage to support-HIV+; Testing-clinical |
| 5 | 31,529 | 176,153 | X | X | X | X | X | X | Community level intervention; Linkage to support-HIV+; PEP HR- |
| 6 | 32,034 | 184,282 | X | X | X | X | X | X | Community level intervention; Linkage to support-HIV+; Testing-clinical; PEP HR- |
| 7 | 33,304 | 294,768 | X | X | X | X | X | X | Community level intervention; Linkage to support-HIV+; Testing-clinical; PEP |

**Additional Sensitivity Analyses**

**Figure S6a,b.** *One way sensitivity analyses for effectiveness and cost-effectiveness ratio of HIV prevention strategies in NYC*. **a.** Range of values for % of HIV averted for each intervention when all input parameters are varied across their spectrum of values that were considered. **b.** range of values for cost-per-infection averted among those interventions found to be cost-saving under reference case assumptions. *Note that STD screening intervention effect size plausible range includes a null effect, therefore, the upper limit of the cost-per-infection averted parameter for this intervention is undefined.*

a.

b.

**Table S6.** Alternate time horizons (5, 10 years) of computer simulation and comparative effectiveness of HIV prevention strategies in NYC

| **Intervention** | **Target Group** | **# infections averted, 5 years** | **Cost per infection averted, 5 years** | **# infections averted, 10 years** | **Cost per Infection averted, 10 years** |
| --- | --- | --- | --- | --- | --- |
| CONDOMS DIST | HIV-infected, high-risk | 397 | $2,544 | 803 | $2,621 |
| SOCIAL MARKETING | HIV-infected | 1,570 | $3,014 | 2,993 | $3,165 |
| CONDOMS DIST | HIV-infected | 616 | $5,557 | 1,232 | $5,572 |
| COMMUNITY INTERVENTION | All | 2,996 | $6,968 | 6,177 | $6,738 |
| TARGETED SURVEILLANCE | HIV-infected | 173 | $24,036 | 332 | $25,215 |
| COFACTORS | HIV-infected, high-risk. | 544 | $29,725 | 1,091 | $29,966 |
| SBIRT | HIV-infected, haz alcohol | 83 | $34,842 | 165 | $35,233 |
| SOCIAL MARKETING | Providers | 2,269 | $79,407 | 4,698 | $76,448 |
| SOCIAL MARKETING | All | 2,996 | $80,174 | 6,177 | $77,530 |
| LINKAGE TO CARE | HIV-infected | 2,114 | $111,350 | 394 | $83,142 |
| SOCIAL MARKETING | HIV-uninfected, high-risk | 151 | $118,891 | 4,534 | $103,492 |
| CONDOMS DIST | HIV-uninfected, high-risk | 745 | $121,311 | 1,492 | $120,618 |
| LINKAGE TO SUPPORT | HIV-infected | 3,488 | $124,908 | 7,004 | $123,932 |
| CONDOMS DIST | All | 840 | $176,921 | 1,682 | $176,188 |
| PARTNER SERVICES | HIV-infected and partners | 104 | $196,085 | 206 | $191,316 |
| STD SCREENING | HIV-infected, high-risk | 264 | $306,349 | 529 | $310,140 |
| STD SCREENING | HIV-infected | 283 | $446,138 | 567 | $447,460 |
| RISK REDUCTION | HIV-infected | 1,570 | $665,922 | 2,993 | $699,261 |
| SOCIAL SERVICES | HIV-uninfected, high-risk | 920 | $1,091,822 | 2,006 | $997,453 |
| CARE COORDINATION | HIV-infected, on ART | 2,616 | $1,187,278 | 5,400 | $1,164,507 |
| TESTING-CLINICAL | HIV uninfected | 968 | $2,112,040 | 2,257 | $1,806,589 |
| TESTING-NON-CLINICAL | HIV-uninfected | 986 | $3,347,741 | 2,133 | $3,083,924 |
| COFACTORS | HIV-uninfected, high-risk | 164 | $3,530,302 | 340 | $3,392,947 |
| SBIRT | HIV-uninfected, high-risk | 36 | $3,769,864 | 75 | $3,626,815 |
| PEP HR(-) | HIV-uninfected, high-risk | 4,819 | $9,217,364 | 9,632 | $9,193,904 |
| STD SCREENING HR(-) | HIV-uninfected, high-risk | 366 | $10,616,972 | 736 | $10,527,979 |
| PEP | HIV-uninfected | 5,244 | $13,660,657 | 10,461 | $13,656,065 |
| STD SCREENING ALL | All | 389 | $16,447,070 | 782 | $16,330,584 |

**Figure S7a,b.** *Effects of optimization by level of evidence*. **a.** Efficient frontier of combinations of HIV prevention strategies filtered by level of evidence. Packages represent combinations of only those strategies that met or exceeded a specified level of evidence and which had an ICER that was of optimal value. All other combinations fall to the right of the curve and are therefore not preferred (and not shown on the figure). Level A ( ) included only those interventions with an evidence grade of A; Level B ( ) included interventions with level of evidence grade A or B; Level C ( ) included interventions with level of evidence grade A or B or C. No optimization curve could be generated for all interventions (i.e. any evidence grade) because of a limitation of computing resources and runtime necessary. **b.** Table which provides details on the packages which lie on the efficient frontier including the specific pathways activated by each package of interventions.

**a.**

|  |  |  | Pathways that are activated by interventions in package | | | | | |  |  |
| --- | --- | --- | --- | --- | --- | --- | --- | --- | --- | --- |
| Package | Number of HIV infections averted over 20 years | Total cost (millions USD) over 20 years | *More consistent condom usage* | *Lower likelihood of having multiple sexual partners* | *Increased likelihood of HIV testing* | *Increased likelihood of linkage to HIV care* | *Increased likelihood of adherence to HIV treatment* | *Increased likelihood of using post-exposure prophylaxis* | Interventions included in package | |
| A1* | 1,514 | $0.4 | X |  |  |  |  |  | Condoms-HR+ | |
| A2* | 2,312 | $1.4 | X |  |  |  |  |  | Condoms-HIV+ | |
| A3 | 3,122 | $372 | X |  |  |  |  |  | Condoms-HIV+; Condoms-HR- | |
| A4 | 3,153 | $590 | X |  |  |  |  |  | Condoms-General | |
| B1 | 3,135 | $71 | X | X |  | X |  |  | Condoms-HIV+; LTC | |
| B2 | 3,777 | $429 | X | X |  | X |  |  | Condoms-HIV+; Condoms-HR-; LTC | |
| B3 | 12,522 | $13,122 | X | X |  | X | X |  | Condoms-HR-; LTC; Care coord | |
| B4 | 12,526 | $13,130 | X | X |  | X | X |  | Condoms-HIV+; Condoms-HR-; LTC; Care coord | |
| B5 | 12,551 | $13,193 | X | X | X | X | X |  | Condoms-HIV+; Condoms-HR-; LTC; Care coord; Partner services | |
| B6 | 14,766 | $21,584 | X | X | X | X | X |  | Condoms-HIV+; Condoms-HR-; LTC; Care coord; Testing-clinical | |
| B7 | 14,778 | $21,641 | X | X | X | X | X |  | Condoms-HIV+; Condoms-HR-; LTC; Care coord; Partner services; Testing-clinical | |
| B8 | 14,794 | $21,859 | X | X | X | X | X |  | Condoms-General; LTC; Care coord; Partner services; Testing-clinical | |
| C1 | 4,054 | $79 | X | X |  |  | X |  | Condoms-HIV+; Cofactors-HIV+ | |
| C2 | 4,908 | $136 | X | X |  | X | X |  | Condoms-HIV+; LTC; Cofactors-HIV+ | |
| C3 | 5,544 | $494 | X | X |  | X | X |  | Condoms-HIV+; Condoms-HR-; LTC; Cofactors-HIV+ | |
| C4 | 12,788 | $13,177 | X | X |  | X | X |  | Condoms-HR-; LTC; Care coord; Cofactors-HIV+ | |
| C5 | 12,792 | $13,186 | X | X |  | X | X |  | Condoms-HIV+; Condoms-HR-; LTC; Care coord; Cofactors-HIV+ | |
| C6 | 12,817 | $13,248 | X | X | X | X | X |  | Condoms-HIV+; Condoms-HR-; LTC; Care coord; Partner services; Cofactors-HIV+ | |
| C7 | 15,033 | $21,640 | X | X | X | X | X |  | Condoms-HIV+; Condoms-HR-; LTC; Care coord; Testing-clinical; Cofactors-HIV+ | |
| C8 | 15,045 | $21,697 | X | X | X | X | X |  | Condoms-HIV+; Condoms-HR-; LTC; Care coord; Partner services; Testing-clinical; Cofactors-HIV+ | |
| C9 | 15,266 | $23,989 | X | X | X | X | X |  | Condoms-HIV+; Condoms-HR-; LTC; Care coord; Partner services; Testing-clinical; Cofactors-HIV+; Cofactors-HIV- | |
| C10 | 28,556 | $197,673 | X | X | X | X | X | X | Condoms-HIV+; Condoms-HR-; LTC; Care coord; Partner services; Testing-clinical; Cofactors-HIV+; PEP HR- | |
| C11 | 23,670 | $199,818 | X | X | X | X | X | X | Condoms-HIV+; Condoms-HR-; LTC; Care coord; Partner services; Testing-clinical; Cofactors-HIV+; Cofactors-HIV-; PEP HR- | |
| C12 | 28,679 | $200,036 | X | X | X | X | X | X | Condoms-General; LTC; Care coord; Partner services; Testing-clinical; Cofactors-HIV+; Cofactors-HIV-; PEP HR- | |
| C13 | 29,874 | $308,469 | X | X | X | X | X | X | Condoms-General; LTC; Care coord; Partner services; Testing-clinical; Cofactors-HIV+; Cofactors-HIV-; PEP | |

**b.**
